# Laser‐Induced Periodic Phase‐Transition of 2D‐MoTe_2_ Nanograting Template for Frequency‐Shift Digital‐SERS Immunoassay of Autoimmune Disease

**DOI:** 10.1002/advs.75366

**Published:** 2026-04-16

**Authors:** Yao Yao, Lulu Cao, Xiaolin Sun, Qiang Wang, Zhiyang Xu, Tianrui Zhai, Yan Zhao, Yijian Jiang, Zhanguo Li, Yinzhou Yan

**Affiliations:** ^1^ School of Physics and Optoelectronic Engineering Beijing University of Technology Beijing China; ^2^ Department of Rheumatology and Immunology Peking University People's Hospital and Beijing Key Laboratory for Rheumatism Mechanism and Immune Diagnosis Beijing China; ^3^ College of New Materials and Chemical Engineering Beijing Institute of Petrochemical Technology Beijing China

**Keywords:** 2D‐transition metal dichalcogenides (2D‐TMDs), autoimmune disease, immunoassay, laser‐induced phase transition, surface‐enhanced raman spectroscopy (SERS)

## Abstract

Fabrication of cross‐scaled ultrasensitive surface‐enhanced Raman scattering (SERS) substrates ranging from nanogaps for efficient localized surface plasmon resonances (LSPRs) to mm^2^‐size for easy operation is challenging. Here, we propose femtosecond‐laser‐induced periodic phase‐transition (*fs*‐LIPPT) of 1T’‐MoTe_2_ nanograting template, inducing Au nanostructures for frequency‐shift digital‐SERS immunoassay. The interference between the incident transverse electric wave and propagation wave in the 2H‐MoTe_2_/SiO_2_‐interlayer waveguide triggers the highly homogeneous periodic 1T’ phase‐transition pattern. The Au nanoparticles (Au*NP*s) are subsequently reduced at the 1T’ regions in chloroauric acid solution, forming a subwavelength Au*NP*s@1T’‐MoTe_2_ nanograting with 295.0 ± 8.1 nm in period. The Fano resonances by coupling the narrow‐band guided‐mode resonances supported in the 1T’‐MoTe_2_/SiO_2_‐interlayer nanograting with the broadband LSPRs in Au*NP*s promote optical localization, achieving the superior performance with a limit of detection in 10^−14^ m and a SERS performance factor of 7.5 × 10^6^ for rhodamine 6G (R6G). A paradigm of frequency‐shift digital‐SERS immunoassay for serological diagnosis is established using the R6G‐labeled Au*NP*s@1T’‐MoTe_2_ nanograting conjugated with immunoglobulin G of rheumatoid arthritis, by which the discrimination accuracy is unexpectedly close to unity. The present work provides a novel protocol for SERS serum immunoassay, opening opportunities for early diagnosis and accurate prognosis of autoimmune diseases by blood test in the future.

## Graphical abstract

A protocol of *fs*‐laser‐induced periodic phase‐transition of 2D‐MoTe_2_ template, inducing Au*NP*s@1T’‐MoTe_2_ nanograting, is developed. The labeled Au*NP*s@1T’‐MoTe_2_ substrate conjugated with specialized immunoglobulin G demonstrates an unexpectedly high discrimination accuracy close to unity for serological diagnosis of rheumatoid arthritis by frequency‐shift digital‐SERS immunoassay. The present work paves a simple way to achieve early diagnosis and accurate prognosis of autoimmune diseases by blood test.

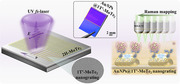

## 1 Introduction

Rheumatoid arthritis (RA) is a systemic autoimmune disease characterized by chronic synovial inflammation, joint destruction, and systemic immune abnormalities [1, 2, 3]. Early diagnosis and accurate prognosis of RA significantly slow down the joint damage and erosive progression [4]. Serological diagnosis of RA primarily relies on anti‐cyclic citrullinated peptide enzyme‐linked immunosorbent assay (ELISA), which is based on a limited set of synthetic citrullinated epitopes, some of which are derived from known antigens, for example, collagen II, vimentin, enolase, etc [5]. More importantly, RA‐associated autoantigens undergo a wide range of protein post‐translational modifications beyond citrullination, including carbamylation (homocitrullination), carboxymethylation, and carboxyethylation, which can generate structurally and antigenically distinct pathogenic neoantigens [6, 7]. Consequently, by focusing on a restricted repertoire of linear epitopes, current ELISA‐based assays exhibit limited sensitivity, particularly in detecting antibodies recognizing complex, differently modified, or conformational epitopes [8]. In addition, the performance of ELISA is highly dependent upon the structural stability and modification status of the antigens, resulting in insufficient coverage of antibody reaction diversity. The method capable of unbiased detection in the interactions between autoantigens and autoantibodies is therefore highly demanded to improve the diagnostic accuracy.

Surface‐enhanced Raman scattering (SERS) is a promising technique for rapid, noninvasive, and nondestructive optical analysis for disease diagnosis [9, 10]. The interaction of molecules with nanostructures is of significance for SERS to reveal the molecular fingerprints from the spectrum [11]. For clinical applications, the SERS substrates are desired with high chemical stability, controllable nanopattern distribution, ease of fabrication, excellent stability, outstanding uniformity, and extraordinary optical responses. Atomically thin 2D transition metal dichalcogenides (2D‐TMDs) have emerged as candidates for semiconductor‐based SERS substrates due to their good homogeneities, high stabilities, and tunable optical properties [12, 13, 14]. They allow uniform chemisorption of molecules, thus facilitating stable and reproducible acquisition of SERS spectra [15]. To overcome the inherent limitations of low chemical enhancement factors of 2D‐TMDs, hybrid substrates comprising metal nanostructures with 2D‐TMDs have been developed. The combination of localized surface plasmon resonances (LSPRs) with chemical enhancement enables ultrasensitive detection down to single‐molecule levels [16]. The universal methods used for depositing highly ordered metal nanostructures on 2D‐TMDs include physical deposition and chemical reduction. The physical deposition typically requires a vacuum or inert atmosphere under elevated ambient temperature, leading to the interfacial stresses and lattice instability of 2D‐TMDs [17]. The chemical reduction usually involves reducing agents interfering with the following SERS detection [18, 19]. Therefore, the fabrication of contamination‐free and highly ordered metal nanostructures on 2D‐TMDs for ultrasensitive SERS is highly demanded.

Femtosecond‐laser‐induced phase‐transition (PT) is a flexible fabrication technique exploiting lattice reconstruction to regulate the properties of 2D‐TMDs [20]. The intense *fs*‐laser pulses reorder the lattice structures in an extremely short time, suppressing the heat‐affected zone to realize PT of 2D‐TMDs from the hexagonal 2H (semiconductor) phase to the octahedral 1T’ (metal) phase [20, 21]. Our previous study has demonstrated the *fs*‐laser‐induced 1T’ phase exhibiting high surface activity enabling in situ reduction of Au nanoparticles (Au*NP*s) [22]. The reduction process was free of reducing agents to avoid contamination. Laser‐induced periodic surface structures (LIPSS) have been witnessed as a general nanofabrication technique [23, 24]. The uniform morphology and period can simply be controlled by wavelength, number of pulses, polarization, fluence, and scanning velocity [25, 26, 27]. However, *fs*‐laser‐induced periodic phase‐transition (*fs*‐LIPPT) in 2D‐TMDs as the template to reduce Au*NP*s for ultrasensitive SERS has not been reported so far.

In this work, we proposed *fs*‐LIPPT in 2H‐MoTe_2_ as a template, followed by solvent‐reducing Au*NP*s for synthesizing large‐area subwavelength Au*NP*s@1T’‐MoTe_2_ nanogratings. The mechanism of *fs*‐LIPPT was revealed theoretically and validated experimentally. The Fano‐shaped absorption in the Au*NP*s@1T’‐MoTe_2_ nanograting on SiO_2_‐interlayer was observed to promote SERS performance. A paradigm of frequency‐shift digital‐SERS (dSERS) immunoassay was also established by conjugation of immunoglobulin G of rheumatoid arthritis (IgG_RA_) with R6G Raman reporter on Au*NP*s@1T’‐MoTe_2_. The rapid and accurate diagnosis of RA was thereby achieved using µL‐volume serum by counting the Raman frequency shift of R6G, which reflects the targeting states of naturally occurring polyclonal autoantibodies toward multiple endogenous autoantigens and their modified forms in serum, enabling unbiased immune profiling without predefined antigen selection. The work provided a straightforward strategy for fabricating cross‐scale homogeneous nanostructures on 2D‐TMDs as ultrasensitive labeled dSERS substrates for the diagnosis of autoimmune diseases by blood test.

## 2 Results and Discussion

### 2.1 Paradigm of Frequency‐Shift dSERS Immunoassay Using Au*NP*s@1T’‐MoTe_2_ Nanogratings

**SCHEME 1:**
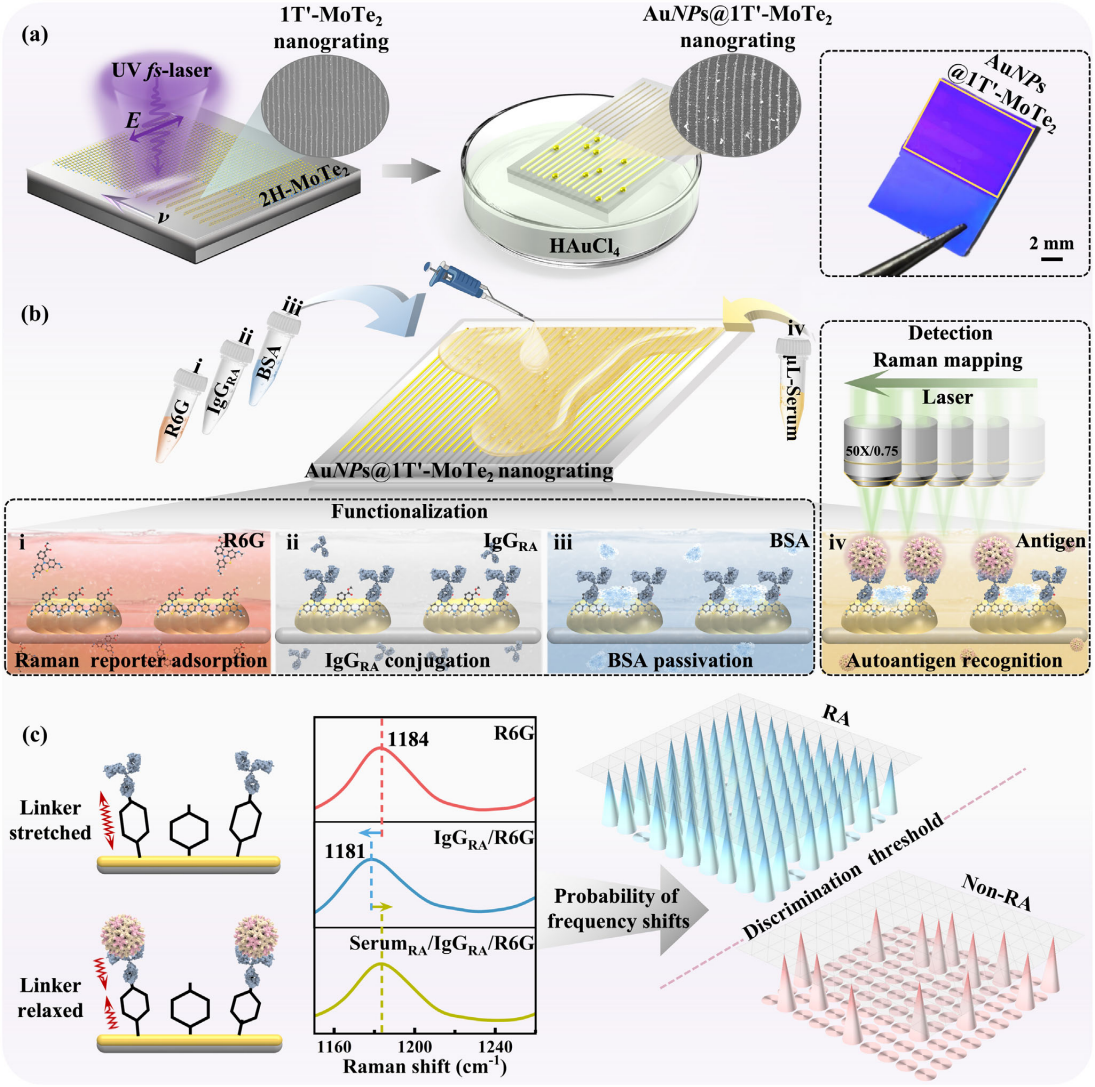
Fabrication of Au*NP*s@1T’‐MoTe_2_ nanograting via *fs*‐LIPPT and paradigm of frequency‐shift dSERS immunoassay. (a) Schematic of *fs*‐LIPPT of 1T’‐MoTe_2_ template and solvent reduction of Au*NP*s for Au*NP*s@1T’‐MoTe_2_ nanograting. (b) Functionalization of Au*NP*s@1T’‐MoTe_2_ nanograting and autoantigen recognition for dSERS analysis. (c) Mechanism of frequency‐shift dSERS immunoassay for serological diagnosis of RA.

Scheme 1a shows the schematic of the *fs*‐LIPPT of the 1T’‐phase nanograting template in 2H‐MoTe_2_ on a SiO_2_/Si substrate. The fabrication was performed in atmospheric ambience using a 343‐nm *fs*‐laser, as shown in Figure S1. The low energy difference (<50 meV) between 2H and 1T’ phases of 2D‐MoTe_2_ facilitated the phase transition by *fs*‐laser irradiation [28]. The spatially periodic distribution during the *fs*‐laser energy deposited onto the 2H‐MoTe_2_ triggered the PT process for 2H/1T’‐MoTe_2_ nanograting morphology (as discussed in the following section). The octahedral 1T’‐MoTe_2_ with high surface activity was then served as a template to reduce the Au*NP*s from HAuCl_4_ solution without any additional reducing agents, by which the Au*NP*s@1T’‐MoTe_2_ nanograting was formed [22]. Scheme 1b demonstrates the frequency‐shift dSERS immunoassay using the Au*NP*s@1T’‐MoTe_2_ nanograting, comprising four steps: (i) adsorption of the rhodamine 6G (R6G) Raman reporters, (ii) conjugation of immunoglobulin G (IgG) isolated from patients’ sera with R6G by amido bonds, (iii) passivation of unbonded sites by bovine serum albumin (BSA), and (iv) recognition of autoantigens in unknown serum for frequency‐shift dSERS immunoassay. The detailed principle of the frequency‐shift dSERS immunoassay is illustrated in Scheme 1c. The linkage of IgG from RA (IgG_RA_) introduced strain stresses to R6G molecules by steric hindrance, by which the R6G structural deformation resulted in the redshift of 1184–1181 cm^−1^ corresponding to the in‐plane C─H bending [29, 30, 31]. The specific targeting of autoantigens by IgG_RA_ released the stresses on R6G, leading to recovery of the frequency shift [32]. The dSERS analysis strategy was employed to evaluate the probability of R6G@1184 cm^−1^ frequency‐shift recovery rate due to the recognition of autoantigens, aiming to distinguish RA and non‐RA controls. It should be noted that the frequency‐shift analysis released the dependence on SERS intensity. It is insensitive to the potential desorption of R6G report molecules during phosphate‐buffered saline (PBS) rinsing in coupling, passivation, and targeting processes. The regions that cannot acquire R6G characteristic Raman signals were set as “disable points” and no longer considered for the following analysis.

### 2.2 *fs*‐LIPPT of 1T’‐MoTe_2_ Template for Au*NP*s@1T’‐MoTe_2_ Nanogratings

**FIGURE 1:**
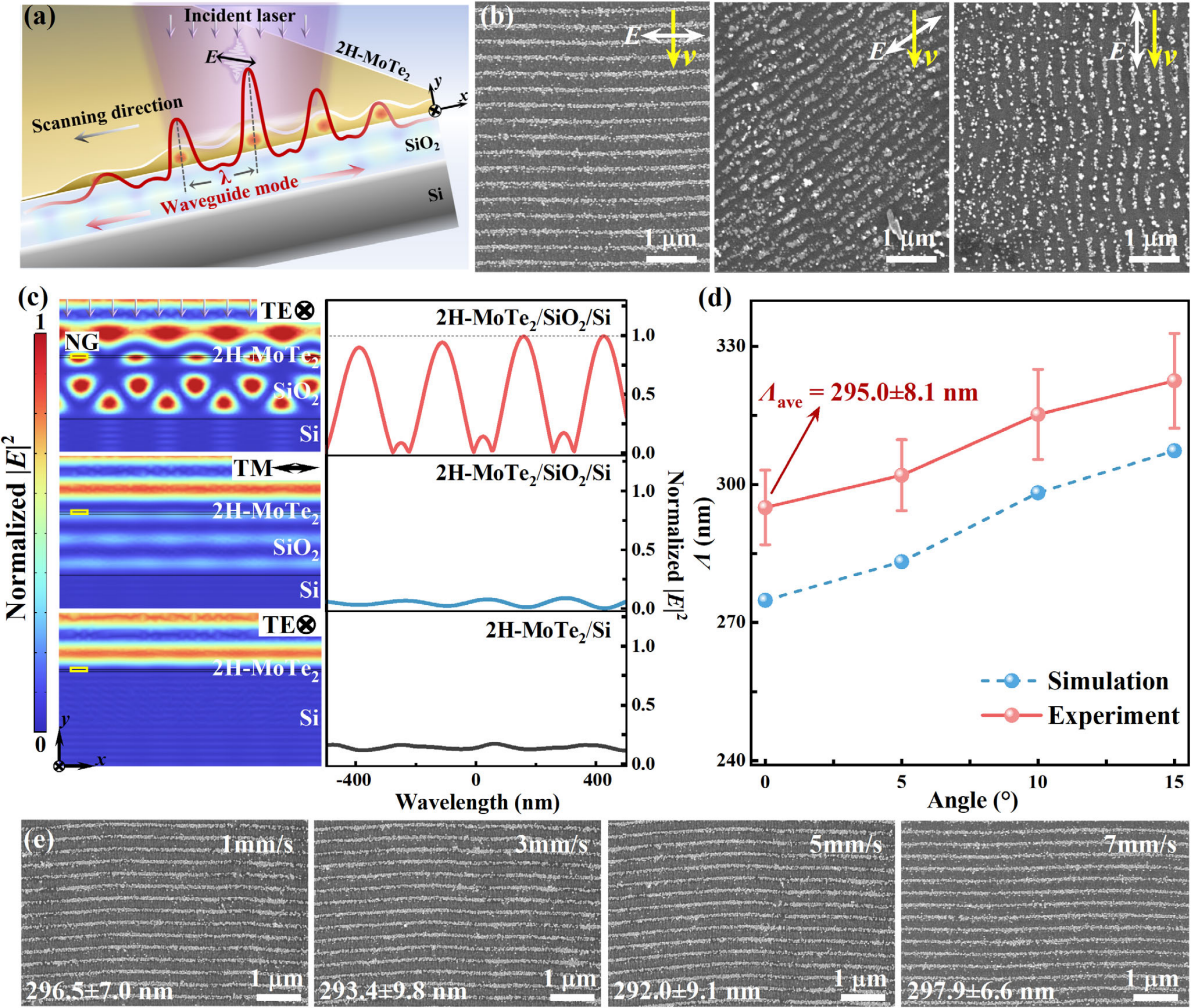
Mechanism of *fs*‐LIPPT of 1T’‐MoTe_2_ nanograting template. (a) Schematic of *fs*‐LIPPT of 1T’‐MoTe_2_. (b) Scanning electron microscope (SEM) micrographs of Au*NP*s@1T’‐MoTe_2_ nanogratings under different polarizations with respect to the scanning direction. (c) Numerical simulation of electric field distribution by interference of waveguide mode in SiO_2_ interlayer with the incident light with various polarizations, where the absence of the SiO_2_ layer is used as the control, and NG represents nanograting. (d) Evolution of laser incident angle with grating period by numerical calculation and experimental measurement. (e) Independence of scanning velocity on the grating period.

The mechanism of *fs*‐LIPPT of 1T’‐MoTe_2_ is shown in Figure 1a. Unlike the low‐spatial‐frequency LIPSS reported in literature [33, 34, 35], the spatial orientation of *fs*‐LIPPT was parallel to the polarization of the incident laser regardless of the scanning direction, as shown in Figure 1b. The optimal morphology of nanograting was realized under the transverse electric (TE) setup with the scanning direction perpendicular to the grating fringe. It should be noted that the SiO_2_ interlayer with a 273 nm thickness under the 2H‐MoTe_2_ was essential to *fs*‐LIPPT formation. In the absence of the SiO_2_ interlayer, the nanograting cannot be formed as Figure S2. It indicated that the spatially periodic energy distribution originated from the interference between incident TM waves and the waveguide modes in the SiO_2_ interlayer with out‐of‐plane polarization. Then the *fs*‐laser‐induced 1T’‐MoTe_2_ nanograting provided extra wavevectors to the following incident light for efficient coupling of waveguide modes in SiO_2_ interlayer to continue the *fs*‐LIPPT process with laser beam scanning.

To validate the above assumption, the light energy distribution was simulated as shown in Figure 1c, where one of the 1T’‐nanogratings was used to scatter the incident light for waveguide mode coupling into the SiO_2_ interlayer. It can be clearly observed that the incident TE wave generated a significant periodic energy distribution due to interference with the waveguide modes supported in the SiO_2_ interlayer. The period (*Λ* = 275 nm) was in agreement with the experimental result (*Λ_ave_* = 295.0 ± 8.1 nm). Neither the transverse Magnetic (TM) incident wave nor the lack of a SiO_2_ interlayer can form the periodic energy distribution under the same superposition configuration. To further highlight the role of waveguide modes in the SiO_2_ interlayer, the effect of incident angle on the nanograting periods was investigated, as Figures 1d (raw data in Figure S3). The oblique incidence provided a phase difference at the wavefronts on the 2H‐MoTe_2_ surface, thereby increasing the grating period. The consistency in trend between numerical simulation and experiment confirmed the proposed interference mechanism of 1T’‐MoTe_2_ nanograting formation via *fs*‐LIPPT. The main advantage of the interference‐induced periodic energy distribution was independent of the laser scanning velocity, as shown in Figure 1e, where the homogeneous nanograting morphology with the constant period was obtained.

**FIGURE 2:**
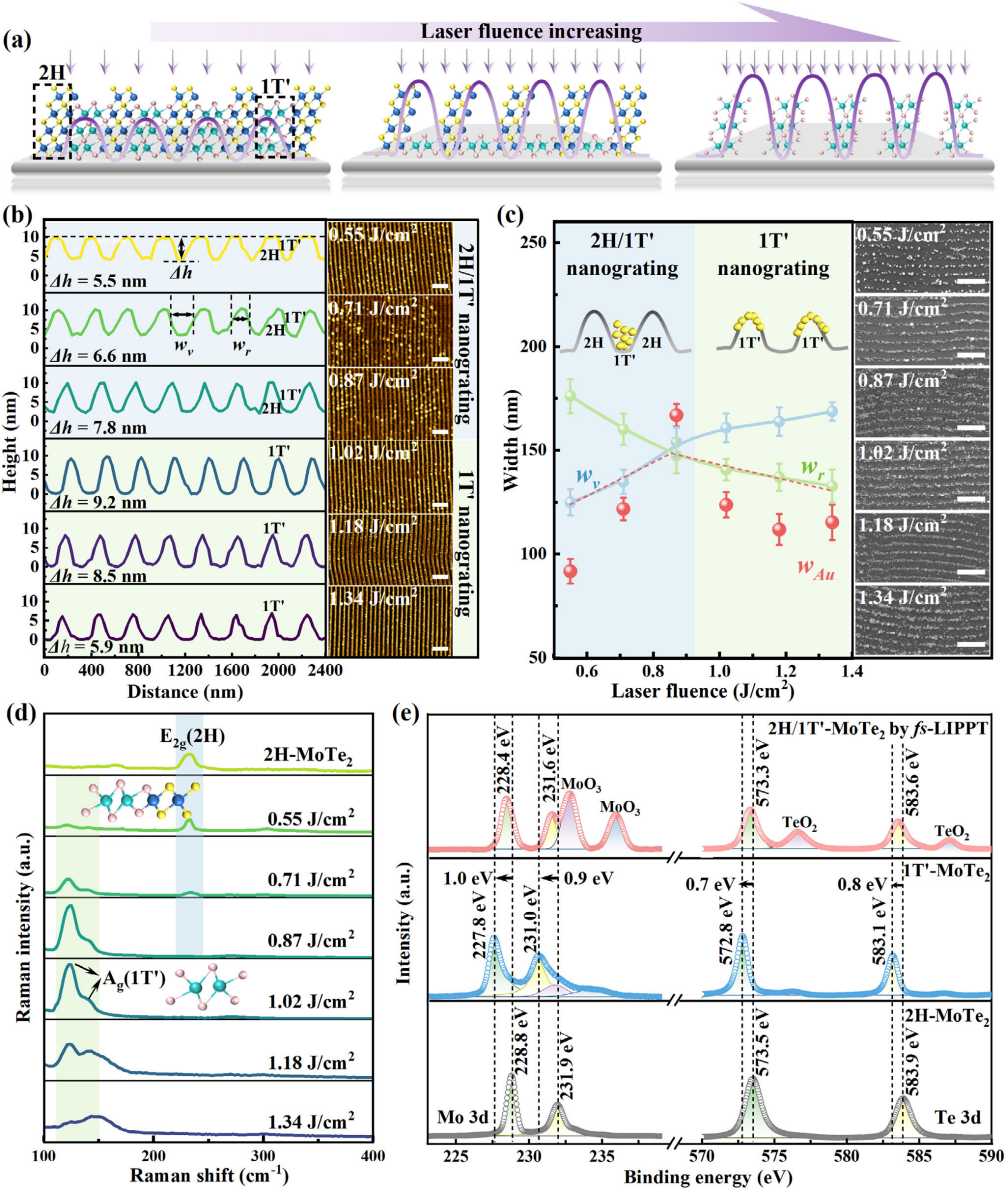
Effect of laser fluence on *fs*‐LIPPT of 1T’‐MoTe_2_ nanograting template. (a) Schematic of *fs*‐LIPPT of 1T’‐MoTe_2_ under different laser fluences. (b) Atomic force microscope (AFM) morphologies of 2H/1T’‐MoTe_2_ and 1T’‐MoTe_2_ nanograting, where the scale bars represent 1 µm. (c) Evolution of 1T’‐MoTe_2_ nanograting ridge width (*w_r_*) and valley width (*w_r_*), as well as the variation of reduced Au*NP*s ridge width (*w_Au_*) with fluence, where the scale bars represent 1 µm. (d) Raman spectra of *fs*‐LIPPT of 1T’‐MoTe_2_ with varied fluences in 0–1.34 J/cm^2^. (e) X‐ray photoelectron spectroscopy (XPS) spectra of Mo 3d and Te 3d orbitals in as‐grown 2H‐MoTe_2_, annealed 1T’‐MoTe_2_, and 2H/1T’‐MoTe_2_ by *fs*‐LIPPT.

It has been acknowledged that the *fs*‐LIPPT from 2H‐ to 1T’‐phase in MoTe_2_ originated from the structural symmetry breaking and ablation thinning by high‐density electron excitation [36]. The spatially periodic energy distribution on the 2H‐MoTe_2_, therefore, induced the 1T’‐phase nanograting with the same period as shown in Figure 2a. The increased laser fluence continued the PT region thinning with a higher contrast, and the extremely high fluence would completely ablate the 1T’‐MoTe_2_ at the constructive zone of interference, whereas the PT process occurred at the destructive zone, resulting in the flipping over of the 1T’‐phase nanograting. The evolution of laser fluence with PT nanograting morphology is demonstrated in Figure 2b. The thickness of the as‐grown 2H‐MoTe_2_ with 15 layers was ∼10 nm [22]. The laser fluence lower than 0.87 J/cm^2^ formed a 2H/1T’ hybrid nanograting, where the 1T’‐phase fringe was continuously thinned and widened with increasing fluence. Further increasing the fluence resulted in the complete ablation of 1T’‐MoTe_2_ at the interfering constructive zone and PT of 2H‐MoTe_2_ at the destructive zone, where the nanograting height (*Δh*) and ridge width were both reduced. Figure 2c confirmed the conversion of the hybrid 2H/1T’‐MoTe_2_ into 1T’‐MoTe_2_ nanograting by reduction of Au*NP*s under various fluences. The ridge width of Au*NP*s (*w_Au_*) initially followed the increase of 1T’‐phase valley width (*w_v_*) in the 2H/1T’‐MoTe_2_ nanograting below 0.87 J/cm^2^, as indicated by the red dashed line in Figure 2c. When the laser fluence exceeded 1.02 J/cm^2^, *w_Au_* was reduced following the variation of nanograting ridge width (*w_r_*), indicating the pure 1T’ phase MoTe_2_ nanograting formed.

Figure 2d shows the Raman spectra of 2H/1T’‐MoTe_2_ by *fs*‐LIPPT from 0 to 1.34 J/cm^2^. The characteristic Raman shifts at 235 cm^−1^ was corresponded to the E_2g_ mode in 2H‐MoTe_2_, whereas the 124 cm^−1^ and 138 cm^−1^ were related to the A_g_ modes in 1T’‐MoTe_2_ [37]. The coexistence of E_2g_ and A_g_ modes confirmed the hybrid 2H/1T’‐MoTe_2_ induced by the fluence in 0.55–0.87 J/cm^2^. The high fluence resulted in the increased ratio of 1T’ to 2H phases, and then the 1T’‐MoTe_2_ regions were continuously thinning. The vanished E_2g_ mode indicated the complete transition to the 1T’ phase under the fluence greater than 1.02 J/cm^2^. The X‐ray photoelectron spectroscopy (XPS) spectra of Mo 3d and Te 3d orbitals in 2H/1T’‐MoTe_2_ fabricated by 0.87 J/cm^2^ are shown in Figure 2e, in which the as‐grown 2H‐MoTe_2_ and annealed 1T’‐MoTe_2_ were used as the standard controls. The Mo 3d_5/2_ and Mo 3d_3/2_ peaks of 2H‐MoTe_2_, located at 228.8 and 231.9 eV, were shifted to 227.8 and 231.0 eV after transition to the 1T’‐phase. Similarly, the Te 3d_5/2_ and Te 3d_3/2_ peaks were redshifted from 573.5 and 583.9 eV to 572.8 and 583.1 eV, respectively. The XPS peaks of 2H/1T’‐MoTe_2_ by *fs*‐LIPPT were located between the 2H‐ and 1T’‐phases, that is, 228.4 eV for Mo 3d_5/2_, 231.6 eV for Mo 3d_3/2_, 573.3 eV for Te 3d_5/2_, and 583.6 eV for Te 3d_3/2_, confirming the formation of hybrid 2H/1T’‐MoTe_2_ nanograting. The 2H/1T’‐MoTe_2_ nanograting fabricated by *fs*‐LIPPT with the fluence of 0.87 J/cm^2^ possessed a high proportion of 2H phase, resulting in the Mo 3d and Te 3d peaks close to the as‐grown 2H‐MoTe_2_.

### 2.3 Fano‐Resonances in Au*NP*s@1T’‐MoTe_2_ Nanograting for Optical Localization Promotion

**FIGURE 3:**
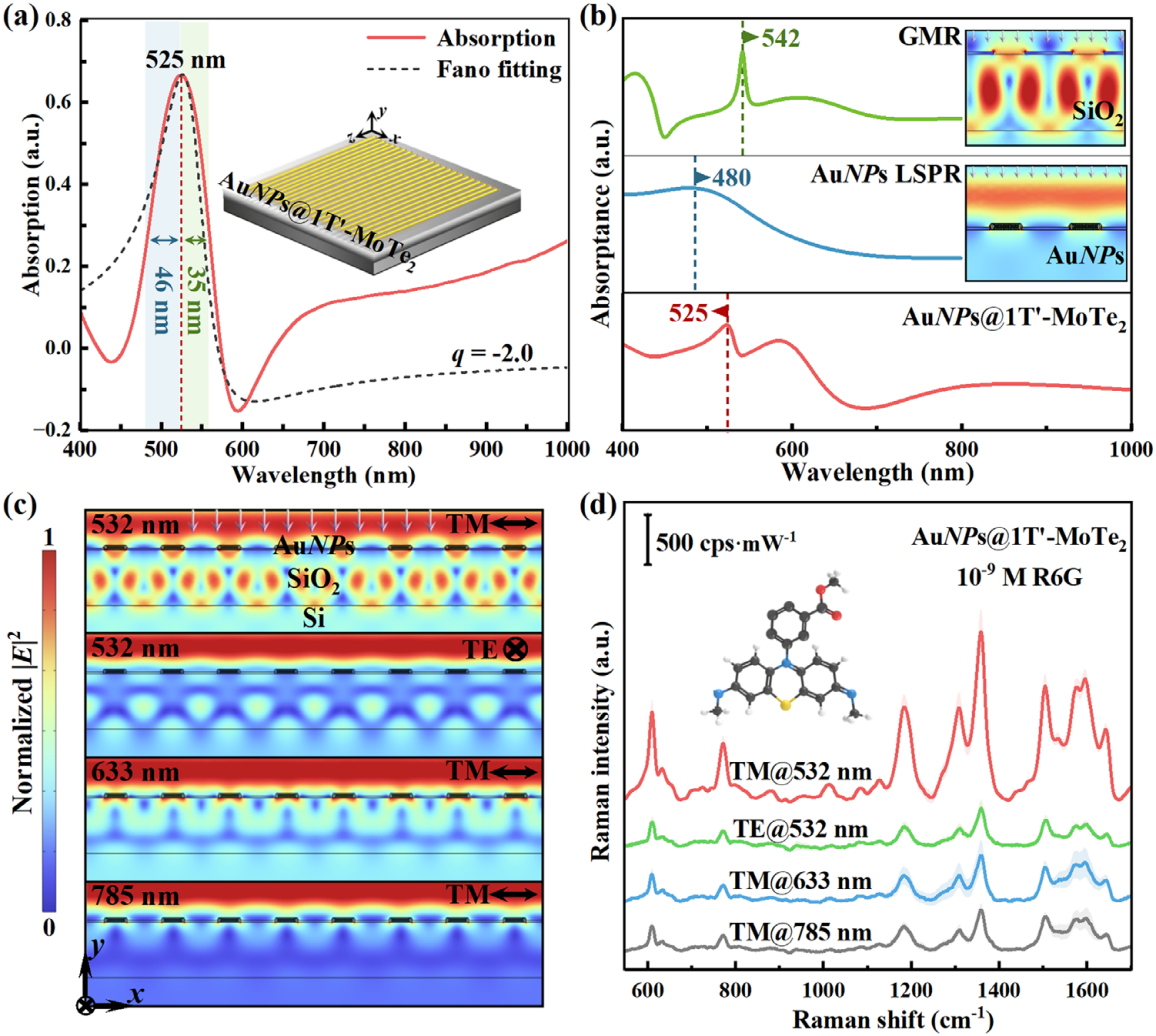
Fano‐resonances in Au*NP*s@1T’‐MoTe_2_ nanograting. (a) Asymmetric absorption of an Au*NP*s@1T’‐MoTe_2_ nanograting, fitted by a Fano line‐shape. (b) Calculated absorptance spectra promoted by GMRs in 1T’‐MoTe_2_ nanograting with SiO_2_ interlayer, LSPRs in Au*NP*s, and Fano resonances in Au*NP*s@1T’‐MoTe_s_ nanograting. (c) Electric field distributions under various incident polarizations and wavelengths on/off Fano resonances. (d) SERS spectra of 10^−9^ m R6G on Au*NP*s@1T’‐MoTe_2_ nanograting under different excitation conditions for on/off Fano‐resonances.

The absorption spectrum representing the optical localization in Au*NP*s@1T’‐MoTe_2_ nanograting is shown in Figure 3a, where the raw reflectance spectra are referred to in Figure S4. The absorption band was located at 525 nm with an asymmetric Fano line‐shape (*q* = −2.0), indicating strong resonances occurring in the Au*NP*s@1T’‐MoTe_2_ nanograting. To elucidate the origin of the Fano line‐shape absorption, the contribution of Au*NP*s@1T’‐MoTe_2_ nanograting with different configurations to absorption was calculated as Figure 3b. Owing to the subwavelength structure, the incident light was diffracted with an extra horizontal vector, that is, *|* ***k*** *|* = 2π/*Λ* (where *Λ* is the grating period), provided by the nanograting. The incident TM waves were hence trapped by the nanograting/SiO_2_‐interlayer waveguide for guided‐mode resonances (GMRs) when the propagation constant matched the specific mode condition [38, 39, 40, 41]. A narrow resonant absorption band appeared at 542 nm, as shown in the top panel of Figure 3b. The Au*NP*s on the nanograting provided a typical broadband LSPR absorption band at 480 nm, as shown in the middle panel of Figure 3b. The coupling between the narrow‐band GMRs and the broadband LSPRs with the in‐plane polarization generated the asymmetric Fano line‐shape absorption at 525 nm, as illustrated in the bottom panel of Figure 3b, facilitating efficient confinement for strong light‐matter interaction in the near field of Au*NP*s@1T’‐MoTe_2_ nanograting [42].

Figure 3c shows the electric field distributions in Au*NP*s@1T’‐MoTe_2_ nanograting under different incident light polarizations and wavelengths. The incident TM wave with near‐resonance wavelength at 532 nm excited the Fano resonances owing to coupling GMRs with LSPRs, by which the incident light was highly confined in the Au*NP*s@1T’‐MoTe_2_ nanograting. It should also be noted that neither a TE wave nor an off‐resonance wavelength wave can achieve Fano resonances. The contribution of Fano resonances in the nanograting for SERS is further demonstrated in Figure 3d, using 10^−9^ m R6G as the calibration. It can be clearly seen that the SERS intensity undergoing Fano resonances was superior to other off‐resonance conditions.

### 2.4 Robustness of Au*NP*s@1T’‐MoTe_2_ Nanogratings for SERS

**FIGURE 4:**
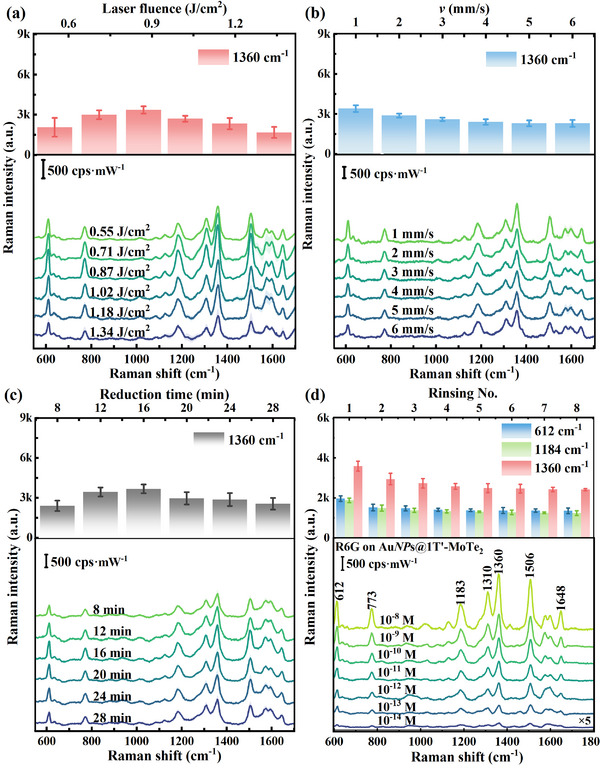
Robustness of Au*NP*s@1T’‐MoTe_2_ nanograting for SERS. SERS performances of Au*NP*s@1T’‐MoTe_2_ nanograting by various (a) *fs*‐laser fluences, (b) scanning velocity, and (c) solvent reduction times. (d) SERS spectra of R6G with concentrations varied from 10^−8^ to 10^−14^ m and the SERS intensity variations after multiple PBS rinsing.

The tolerance of fabrication parameters for Au*NP*s@1T’‐MoTe_2_ nanograting is of importance in SERS applications. Figure 4a‐c illustrates the effects of laser fluence, scanning velocity, and solvent reduction time on SERS performance. There existed a large parameter window for *fs*‐LIPPT fabrication of Au*NP*s@1T’‐MoTe_2_ nanograting, where the laser fluence in 0.55–1.34 J/cm^2^, the scanning velocity in 1–6 mm/s, and the solvent reduction time greater than 8 min facilitated the achievement of reproducible and stable SERS spectra. The parameter set of fluence of 0.87 J/cm^2^ with scanning velocity of 1 mm/s, followed by 16 min reduction in HAuCl_4_ solution, was chosen for the following investigation. The homogeneity of a fabricated SERS substrate was characterized via Raman mapping of 9 typical 20 × 20 µm^2^ regions across a 10 mm^2^ area, as shown in Figure S5. The mean relative standard deviation (RSD) from the 9 regions was 8.3 % ± 0.8 %. Figure 4d demonstrates the limit of detection (*LoD*) down to 10^−14^ m for R6G with the SERS performance factor (*SPF*) up to 7.5 × 10^6^ (see Section 4). Further considering the clinical applications as discussed in the following section, where the PBS rinsing is an essential step to minimize the non‐specific binding during SERS immunoassay, we examined the variation of R6G SERS intensity on Au*NP*s@1T’‐MoTe_2_ nanograting after up to 8 rinsing cycles, as shown in the upper panel of Figure 4d. The slightly decreased intensities of characteristic Raman shifts at 612, 1184, and 1360 cm^−1^ demonstrated good durability in rinsing cycles. It should also be noted that the dSERS analysis only monitored the frequency shifts rather than the Raman intensities of R6G. The region's lack of R6G characteristic Raman peaks caused by desorption of reporter molecules was set as a disable point and removed to eliminate potential analysis errors.

### 2.5 Frequency‐Shift dSERS Immunoassay for RA Diagnosis

**FIGURE 5:**
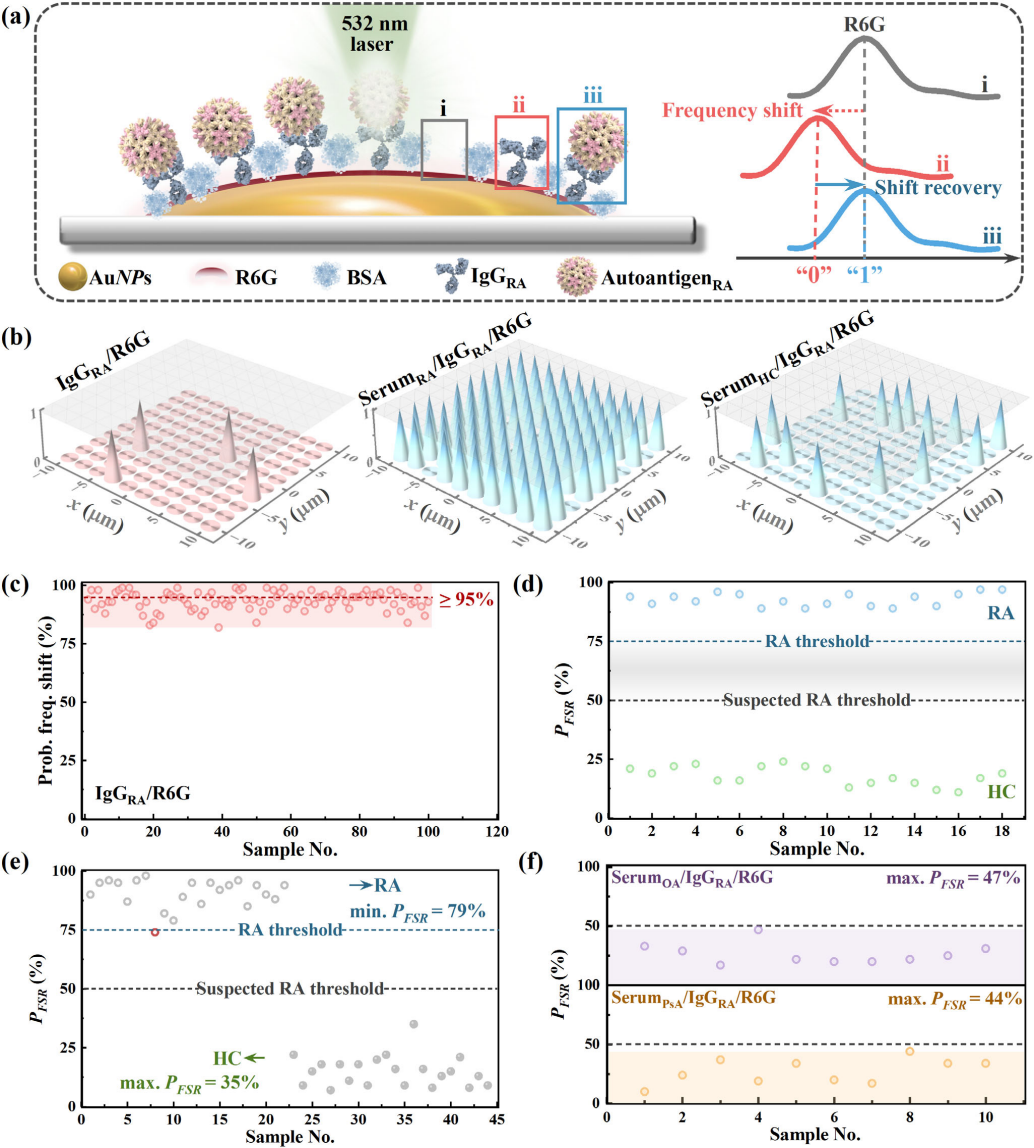
Frequency‐shift dSERS immunoassay using Au*NP*s@1T’‐MoTe_2_ nanogratings. (a) Schematic of frequency‐shift dSERS immunoassay by IgG_RA_/R6G‐labeled Au*NP*s@1T’‐MoTe_2_ nanograting for diagnosis of RA using serum. (b) Frequency‐shift dSERS analysis of serum on IgG_RA_/R6G‐labeled Au*NP*s@1T’‐MoTe_2_ nanogratings. (c) Probability of frequency shift for 100 substrates (with 10 × 10 positions each) of Au*NP*s@1T’‐MoTe_2_ nanogratings to confirm the efficiency of IgG_RA_ conjugation. (d–f) *P_FSR_* distribution and dual threshold discrimination criterion of (d) RA and HC sera, (e) diagnosis for blind‐test group, and (f) cross‐reactivity validation using the frequency‐shift dSERS immunoassay.

The Au*NP*s/1T’‐MoTe_2_ nanograting labeled with R6G Raman reporters provided a powerful tool for discrimination of RA by trace serum down to 2.5 µL. The principle of frequency‐shift dSERS immunoassay is illustrated in Figure 5a. The IgG isolated from 40 RA patients’ sera (IgG_RA_) functionalized the R6G‐labeled Au*NP*s/1T’‐MoTe_2_ nanograting via activation of 1‐ethyl‐3‐(3‐dimethylaminopropyl) carbodiimide (EDC)/N‐hydroxysuccinimide (NHS), forming stable amide bonds at the carboxyl‐terminal of IgG_RA_ and the diethylamino of R6G [43]. The conjugation of IgG_RA_ and R6G resulted in the strain stresses on R6G molecules, leading to the redshift of the characteristic peak at 1184–1181 cm^−1^ (as i to ii in Figure 5a). The polyclonal autoantibodies of IgG_RA_ targeted diverse autoantigens and their post‐translationally modified forms in RA serum [6, 7, 44], which recovered the Raman peak back to 1184 cm^−1^ by the stress releasing (as ii–iii in Figure 5a) [29, 45]. By statistically analyzing the frequency shift differences of Raman peaks in 100 spectra of R6G and IgG_RA_/R6G on the Au*NP*s@1T’‐MoTe_2_, the threshold of frequency shift and recovery was set as 1.2 cm^−1^, as shown in Figure S6. For more intuitive comparison, the recovery of frequency shift from 1181 to 1184 cm^−1^ was set as positive “1” for targeting autoantigens; otherwise was negative ∜0∝. The RA diagnosis can therefore be realized by frequency‐shift dSERS analysis, that is, counting the probability of frequency shift recovery (*P_FSR_*) of the Raman peak of R6G, as shown in Figure 5b, in which 10 × 10 random positions were mapped for reliable analysis. Figure 5c displays the frequency shift probability across 100 substrates (with 10 × 10 positions each) of Au*NP*s@1T’‐MoTe_2_ nanogratings labeled with IgG_RA_/R6G. To minimize the signal interference from uncoupled R6G molecules, the IgG_RA_/R6G‐labeled substrates with the probability of frequency shift >95 % were selected for the subsequent experiment.

The discrimination threshold was then evaluated using sera from 18 RA patients and 18 healthy controls (HC) as Figure 5d (raw data in Figure S7), where the sample sizes were determined by stabilization of mean *P_FSR_* as Figure S8a. The RA sera containing multiple autoantigens targeted by IgG_RA_ caused a high *P_FSR_*. In contrast, HC sera cannot recover the Raman frequency due to a lack of specific autoantigens. It can be seen that the *P_FSR_* of RA and HC sera were completely separated with a great difference. Further considering the tolerance of statistical error and readability for clinical diagnosis, we employed a dual threshold strategy for identification of RA sera, in which *P_FSR_* >75 % represented the RA positives with strong confidence and *P_FSR_* in 50 %–75 % indicated the suspected RA with high risk requiring further evaluation. The dual threshold setting originated from the *t*‐test, as Figure S8b, in which the difference in *P_FSR_* between RA and HC sera was highly significant (*p*< 0.001). Figure 5e (raw data in Figure S9) further presents the single‐blind test conducted on 44 RA/HC sera to validate the diagnostic accuracy. The RA patients were completely discriminated with an accuracy unexpectedly to unity by 50 % suspected RA threshold and 97.7 % by 75 % RA threshold criteria, respectively. To evaluate the specificity of the IgG_RA_‐based dSERS immunoassay, cross‐reactivity validation was performed using osteoarthritis (OA) and psoriatic arthritis (PsA) sera. The values of *P_FSR_* were slightly increased to 47 % and 44 %, as Figure 5f (raw data in Figure S10), attributing to the presence of components in OA and PsA sera that can be targeted by the polyclonal autoantibodies of RA. However, the values were still lower than the suspected RA threshold at 50 % demonstrated the superior specificity, where the significant differences between RA and non‐RA sera (*p*< 0.001) were further validated in Figure S8b. Therefore, the IgG_RA_/R6G‐labeled Au*NP*s@1T’‐MoTe_2_ nanograting SERS platforms exhibited outstanding capability for RA diagnosis by µL‐volume serum.

## 3 Conclusions

A protocol of *fs*‐LIPPT of 1T’‐MoTe_2_ as a template achieving Au*NP*s@1T’‐MoTe_2_ nanograting for dSERS immunoassay was developed in this work. The interference of incident TE and horizontally‐propagating waves in 2H‐MoTe_2_/SiO_2_‐interlayer waveguide was confirmed to trigger the 1T’ phase‐transition with the homogeneous spatial period of 295.0 ± 8.1 nm. The solvent‐reduced Au*NP*s@1T’‐MoTe_2_ nanograting with a subwavelength period supported Fano resonances by coupling GMRs with LSPRs in the Au*NP*s@1T’‐MoTe_2_ nanograting for promoting SERS sensitivity with a *LoD* down to 10^−14^ m with a *SPF* of 7.5 × 10^6^ for R6G reporter molecules. The frequency‐shift dSERS immunoassay for serological diagnosis was established by conjugation of IgG_RA_ on the R6G‐labeled Au*NP*s@1T’‐MoTe_2_ nanograting, enabling to target polyclonal autoantigens for complete discrimination of RA from HC and non‐RA autoimmune diseases by µL‐volume serum. This work paved a new strategy for immunoassay of human serum, holding the promise of autoimmune diseases by blood test for future precise treatment.

## 4 Methods

### 4.1 Fabrication of Au*NP*s/1T’‐MoTe_2_ Nanograting

The Au*NP*s@1T’‐MoTe_2_ nanograting were fabricated by the following procedures: (i) *fs*‐LIPPT of 1T’‐MoTe_2_ nanograting template: a CVD‐grown 2H‐MoTe_2_ on SiO_2_/Si (Shenzhen 6Carbon Technology Co., Ltd., China) placed on a motorized linear stage was scanned by a focused 343 nm *fs*‐laser (with a repetition rate of 5 kHz, Light Conversion PH2‐20W‐SP, Lithuania) to induce a periodic 1T’‐PT pattern. A half‐wave plate and a neutral density filter were used to regulate the *fs*‐laser polarization and power, respectively. The laser beam was focused by an objective of 10×/NA0.25, yielding a spot size of 6 µm. (ii) Solvent reduction of Au*NP*s: the *fs*‐fabricated 1T’‐MoTe_2_ nanograting template was immersed in HAuCl_4_ solution (1 mM) for 4–28 min for the reduction of Au*NP*s on 1T’‐phase regions to achieve the Au*NP*s@1T’‐MoTe_2_ nanograting. (iii) The nanograting was rinsed with deionized water to remove the residual solvent.

### 4.2 Characterization

The microstructural morphologies were captured by SEM (Quanta 650 FGE, USA). The surface morphologies of 2H/1T’‐MoTe_2_ nanograting were characterized by AFM (SmartSPM‐1000, Horiba, Japan). X‐ray photoelectron spectroscopy (XPS; NEXSA G2, Thermo Fisher Scientific, USA) was used to analyze the chemical composition and electronic orbitals of the 2H/1T’‐MoTe_2_ before and after *fs*‐LIPPT. The Raman spectra of 2H/1T’‐MoTe_2_ were collected by a SmartRaman *μ*‐Raman system (developed by the Institute of Semiconductors, Chinese Academy of Science) equipped with a high‐resolution spectrometer (Horiba LabRAM iHR550, Japan). The backscattering geometry was configured with a 600 lines/mm grating and a low‐noise CCD. The excitation source was a 633 nm‐line CW laser (manufactured by Thorlabs, Co., Ltd., USA) for Raman spectra of 2H/1T’‐MoTe_2_. The objective of the 50×/NA0.75 focused 2.5 mW laser, and the integration time was set as 5 s. The R6G molecules (Aladdin Holding Group Co., Ltd., China) were used to calibrate the SERS performance of Au*NP*s@1T’‐MoTe_2_ nanograting, which was also exploited as the Raman reporter by labeling onto the nanograting for frequency‐shift dSERS immunoassay. The R6G was first diluted in alcohol solution with various concentrations and then dropped onto Au*NP*s@1T’‐MoTe_2_ nanograting. A 532 nm‐line CW laser (manufactured by Changchun New Industries Technology, Co., Ltd., China) was used as the excitation source matching the LUMO‐HOMO energy of R6G for resonant Raman scattering. The raw beam was focused by the objective of 50×/NA0.75 with a spot size of 0.76 µm, and the Raman spectra were acquired by the same setup of spectrometer as mentioned above. For the dSERS immunoassay, the excitation laser power was fixed at 1.0 m, W and the acquisition time was set to 1s to avoid potential thermal damage of biomolecules. The 2D dSERS mapping with a pitch of 2 µm was realized by a *x‐y* motorized linear stage. The reflectance contrast spectra were acquired using the same spectroscope with a halogen light source (GCI‐0601, Daheng Optics, China).

### 4.3 SERS Performance Factor (*SPF*)

*SPF* is a SERS evaluation parameter independent from the experimental conditions, capable of quantitatively describing the overall contribution to SERS performance [46]. To ensure the relevance and accuracy of the analytical results in relation to the following dSERS immunoassay, the Raman shift of R6G reporter at 1184 cm^−1^ was selected as the calibration for *SPF* calculation. The Raman spectra of R6G on Si were acquired as Figure S11a. The evolution of R6G Raman intensities at 1184 cm^−1^ with concentrations on Si and Au*NP*s@1T’‐MoTe_2_ nanograting were plotted and fitted by the Langmuir isotherm adsorption model, as shown in Figures S11b,c, respectively. The *SPF* of Au*NP*s@1T’‐MoTe_2_ nanograting was therefore determined by [47]:

(1) $$SPF=\frac{\Delta I_{\mathrm{SERS}}}{\Delta C_{\mathrm{SERS}}}/\frac{\Delta I_{\mathrm{Raman}}}{\Delta C_{\mathrm{Raman}}}$$

where $\frac{\Delta I_{\mathrm{SERS}}}{\Delta C_{\mathrm{SERS}}}$ and $\frac{\Delta I_{\mathrm{Raman}}}{\Delta C_{\mathrm{Raman}}}\;$ are the slopes of the fitted curves of Raman intensity with R6G concentration from Au*NP*s@1T’‐MoTe_2_ nanograting and Si substrates, that is, 2.92 × 10^12^ and 3.91 × 10^5^, respectively. *SPF* was hence calculated to be 7.5 × 10^6^.

### 4.4 Numerical Simulation

The numerical simulation of total electric‐field distribution was performed using the COMSOL Multiphysics software package (licensed by COMSOL Co., Ltd.) to reveal the mechanisms of *fs*‐LIPPT and Fano‐resonances. The geometric models were set to be the same as the experiments, as shown in Figure S12. For *fs*‐LIPPT, the 343 nm TE and TM planewaves were employed as illustrated in Figure S12a. A series of 2D cross‐sectional models with various configurations was developed according to the study on electromagnetic distribution in Au*NP*s@1T’‐MoTe_2_ nanogratings for on/off Fano resonances, as shown in Figure S12b. For Fano‐shape absorption in AuNPs@1T’‐MoTe_2_ nanograting, the TE and TM planewaves with 532, 633, and 785 nm wavelengths were used. Air (*n* = 1) was set as the ambience. The refractive indices of Au, Si, and 2H‐MoTe_2_ in the COMSOL material library were utilized, originating from the literature [48, 49, 50]. The refractive index of SiO_2_ was calculated based on the UV–vis spectrum and the thickness identified by depth‐dependent XPS, as detailed in Figure S13. The periodic boundary conditions were applied to the sides of the model for the periodic structures. The perfect matching layers were utilized at the top and bottom boundaries, suppressing the light scattering.

### 4.5 Patients and Serum Samples

The serum samples were collected from 100 sex‐ and age‐matched individuals, including 40 RA patients, 10 OA patients, 10 PsA patients, and 40 HC at Peking University People's Hospital, China. Ethical approval for the study was granted by the Research Ethics Committee of Peking University People's Hospital, and written informed consent was obtained from all participants (Approval No. 2024PHB262‐001). The diagnosis of RA was in accordance with the 2010 American College of Rheumatology /European League Against Rheumatism (ACR/EULAR) classification criteria. Disease activity in RA patients was assessed using the Disease Activity Score in 28 joints (DAS28), erythrocyte sedimentation rate (ESR), C‐reactive protein (CRP) levels, swollen and tender joint counts, and treatment regimens. The diagnosis of OA was based on the American College of Rheumatology (ACR) classification criteria for knee/hip/hand osteoarthritis, supported by clinical symptoms and radiographic findings (Kellgren–Lawrence grade ≥2). The 2019 ACR/Arthritis Foundation guideline was referred for disease management. The diagnosis of PsA fulfilled the CASPAR (Classification Criteria for Psoriatic Arthritis) criteria. Participants were excluded if they met any of the following criteria: (1) concurrent other autoimmune diseases, (2) active viral or bacterial infection, (3) a diagnosis of malignancy, or (4) pregnancy or lactation. The detailed clinical characteristics of the participants are listed in Table S1.

### 4.6 Purification of Autoantibodies From RA Sera for IgG_RA_

Autoantibodies were isolated from serum samples of RA patients using Protein G agarose (Thermo Fisher Scientific, Cat 89979, USA) according to standard affinity purification procedures. Protein G resin was equilibrated by washing twice with sterile PBS (pH 7.4). The serum samples from 18 clinically confirmed RA patients were pooled in equal volumes to obtain a cohort‐level autoantibody preparation, minimizing inter‐individual variation and providing a representative IgG_RA_ sample for downstream assays. The pooled serum was diluted 1:5 (v/v) in PBS to reduce viscosity and ensure optimal binding. The diluted serum was incubated with the equilibrated Protein G resin for 2 h at 4°C using end‐over‐end rotation. Following incubation, the flow‐through was removed by gravity, and the resin was washed with 5–10 column volumes of PBS to eliminate nonspecifically bound components. Bound IgG_RA_ antibodies were eluted using 0.1 m glycine‐HCl (pH 2.5), and each collected fraction was immediately neutralized with 1 m Tris‐HCl (pH 8.0). Neutralized eluates were pooled, and buffer exchanged into PBS. The antibody concentration was measured via UV absorbance at 280 nm.

### 4.7 Procedure of Frequency‐Shift dSERS Immunoassay

First, the prepared Au*NP*s@1T’‐MoTe_2_ nanograting was immersed in a 10^−9^ m R6G ethanol solution for 1 h to allow sufficient adsorption of R6G. Then, the substrate was thoroughly rinsed 8 times with ethanol and PBS to prevent molecular aggregation and remove ‌unadsorbed R6G. Subsequently, the frozen IgG_RA_, RA, HC, PsA, and OA sera were first retrieved from a −80°C freezer and thawed gradually at 4°C for approximately 20–30 min until fully liquefied. A volume of 10 µL of IgG_RA_ was diluted to 100 µL in PBS (pH 7.4), followed by the addition of EDC/NHS solution (171 mM/427.5 mM). The mixture was activated at room temperature for 40 min. Next, the R6G‐labeled Au*NP*s@1T’‐MoTe_2_ were immersed in the mixed solution and reacted at room temperature for 2 h to ensure adequate conjugation of IgG_RA_ with R6G. After that, the substrate was rinsed with PBS, immersed in a 1 % BSA solution for 30 min to block unbound sites, and rinsed with PBS again, to obtain the IgG_RA_/R6G‐labeled Au*NP*s@1T’‐MoTe_2_. Finally, serum was dropped onto the substrate and rinsed with PBS after 1 h incubation at room temperature for dSERS acquisition.

## Author Contributions

Yao Yao conducted all experiments and numerical simulations and wrote the first draft. Lulu Cao provided serum samples, prepared IgGRA, and revised the manuscript. Xiaolin Sun supervised the experiments on human serum. Qiang Wang conducted the XPS analysis. Zhiyang Xu configured the fs‐laser nanofabrication system. Tianrui Zhai advised on the fs‐laser nanofabrication. Yan Zhao and Yijian Jiang advised on the Raman spectral analysis. Zhanguo Li advised on the clinical diagnosis of autoimmune diseases. Yinzhou Yan supervised the project, provided the original idea, and finalized the manuscript. The manuscript was written through the contributions of all authors.

## Conflicts of Interest

The authors declare no conflicts of interest.

## Supporting information




**Supporting File**: advs75366‐sup‐0001‐SuppMat.docx.

## Data Availability

The data that support the findings of this study are available from the corresponding author upon reasonable request.
