# Supplementary material for: Laser‐Induced Periodic Phase‐Transition of 2D‐MoTe2 Nanograting Template for Frequency‐Shift Digital‐SERS Immunoassay of Autoimmune Disease
Source: Adv Sci (Weinh). 2026 Apr 16;13(39):e75366. doi: 10.1002/advs.75366 (PMC13335738; doi:10.1002/advs.75366)
Supplement: Supplementary file 1 — Supporting File: advs75366‐sup‐0001‐SuppMat.docx. [file ADVS-13-e75366-s001.docx]

**Supporting Information**

Laser-Induced Periodic Phase-Transition of 2D-MoTe_2_ Nanograting Template for Frequency-Shift digital-SERS Immunoassay of Autoimmune Disease

Yao Yao^1^, Lulu Cao^2^, Xiaolin Sun^2^, Qiang Wang^3^, Zhiyang Xu^1^, Tianrui Zhai^1^, Yan Zhao^1^, Yijian Jiang^1^, Zhanguo Li^2,*^, Yinzhou Yan^1,*^

^1^ School of Physics and Optoelectronic Engineering, Beijing University of Technology, Beijing 100124, China

^2^ Department of Rheumatology and Immunology, Peking University People’s Hospital and Beijing Key Laboratory for Rheumatism Mechanism and Immune Diagnosis (BZ0135), Beijing, 100044, China

^3^ College of New Materials and Chemical Engineering, Beijing Institute of Petrochemical Technology, Beijing 102617, China


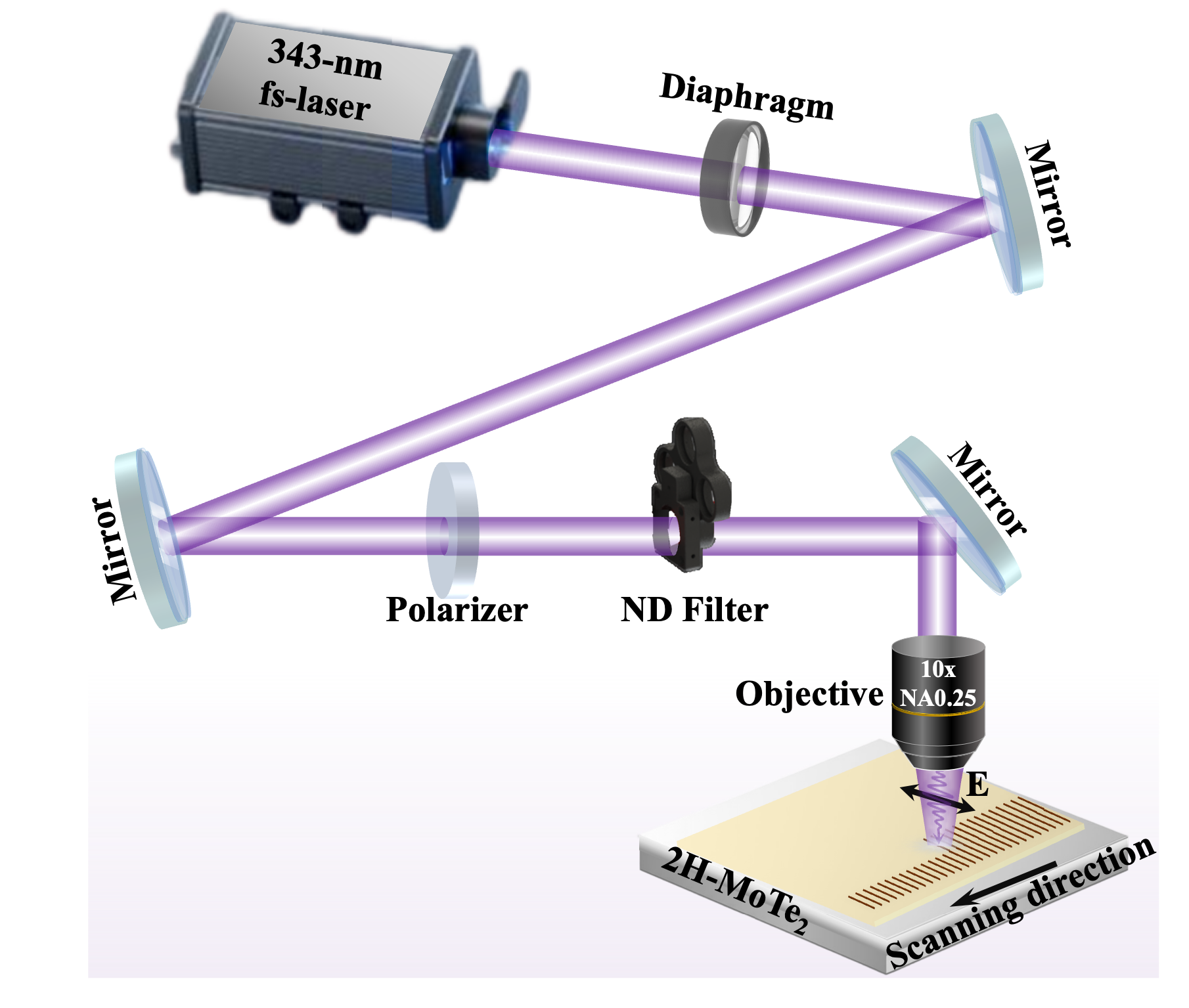


**Figure S1.** Experimental setup of *fs*-LIPPT of 1T’-MoTe_2_ template. ND filter represents the neutral density filter for *fs*-laser power regulation.


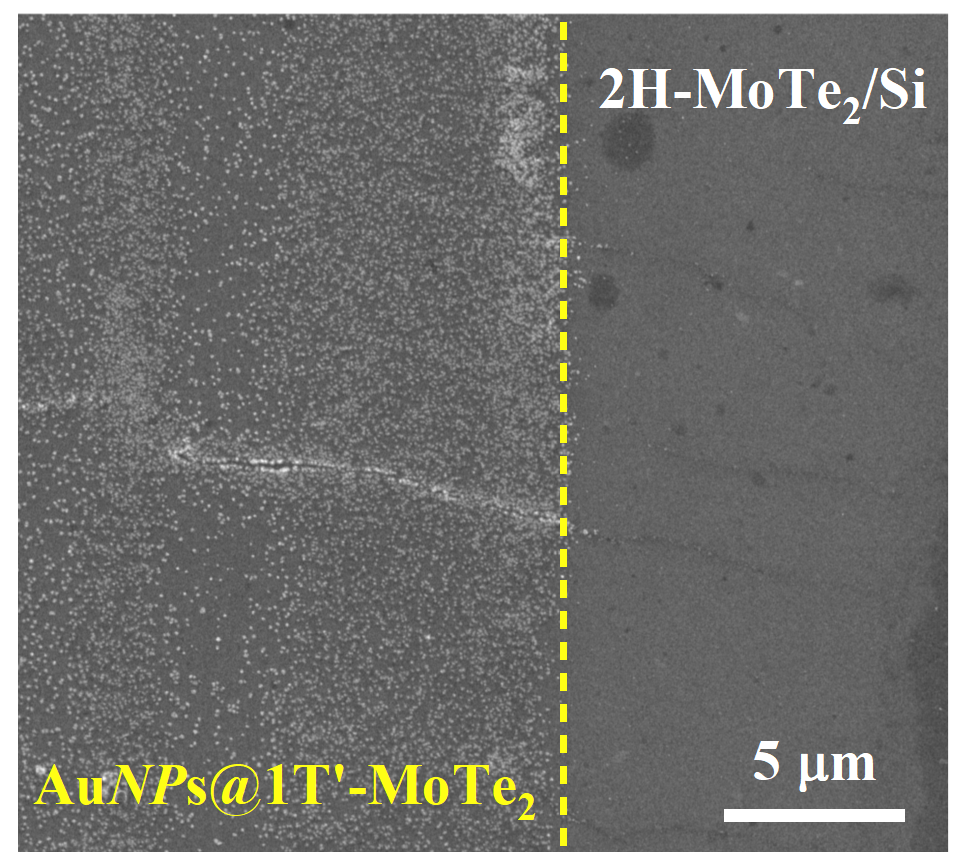


**Figure S2.** *fs*-LIPPT of 1T’-MoTe_2_ in absence of SiO_2_ interlayer followed by reduction of Au*NP*s.


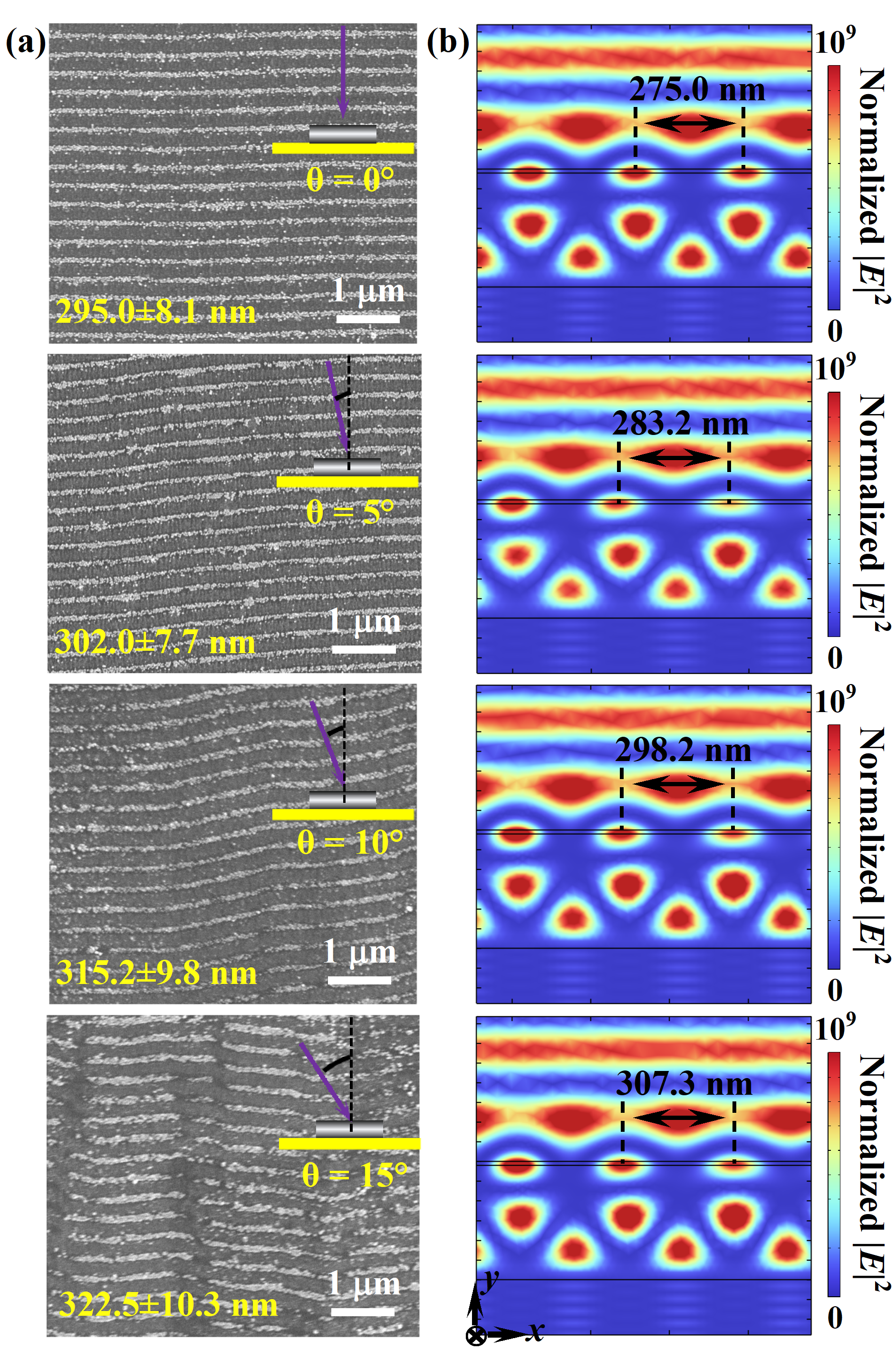


**Figure S3.** Raw data on evolution of laser incident angle with grating period in (a) experiment and (b) numerical simulation.


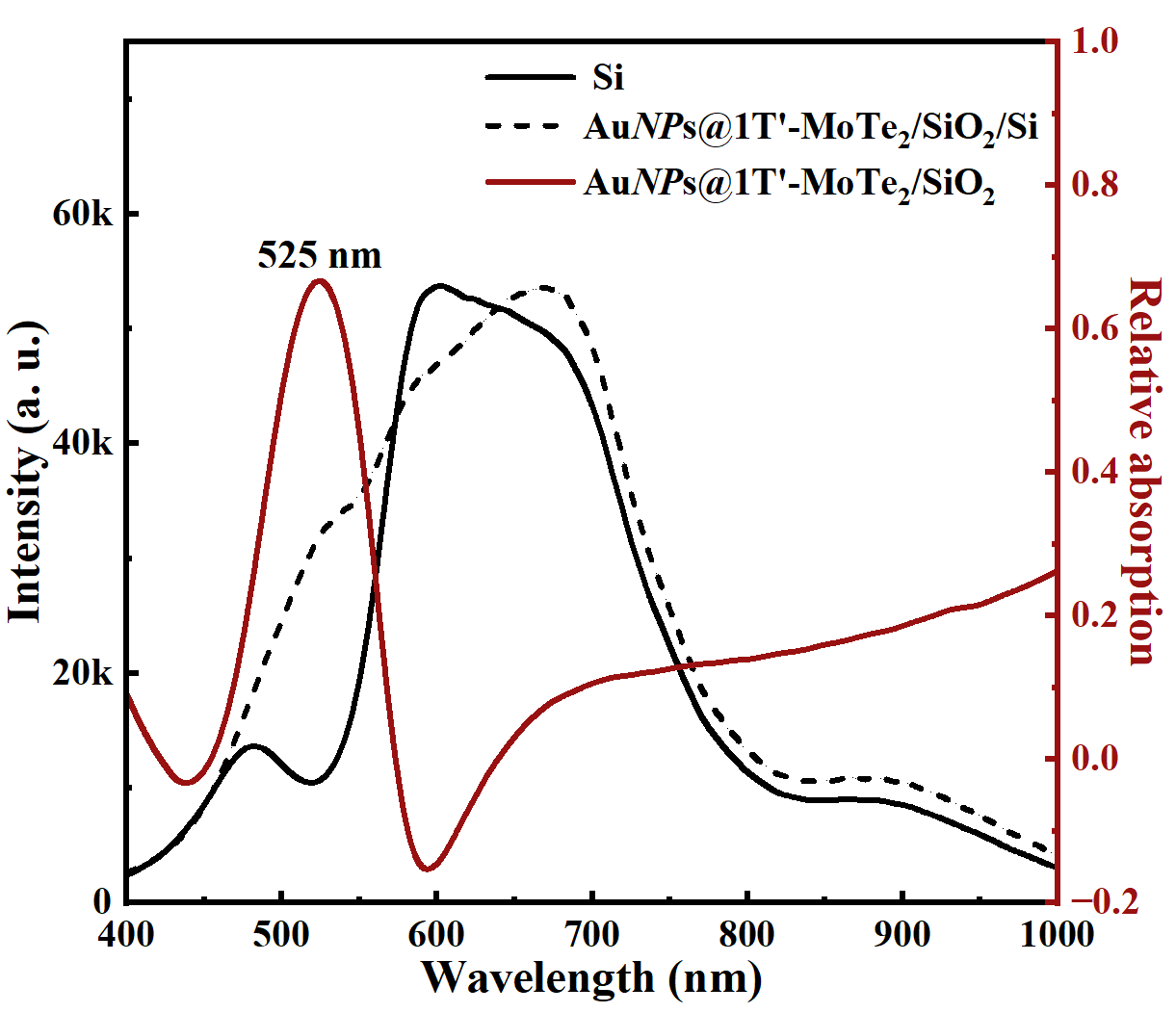


**Figure S4.** Reflectance spectra of Si and Au*NP*s@1T’-MoTe_2_/SiO_2_/Si and contrast spectrum of Au*NP*s@1T’-MoTe_2_/SiO_2_.


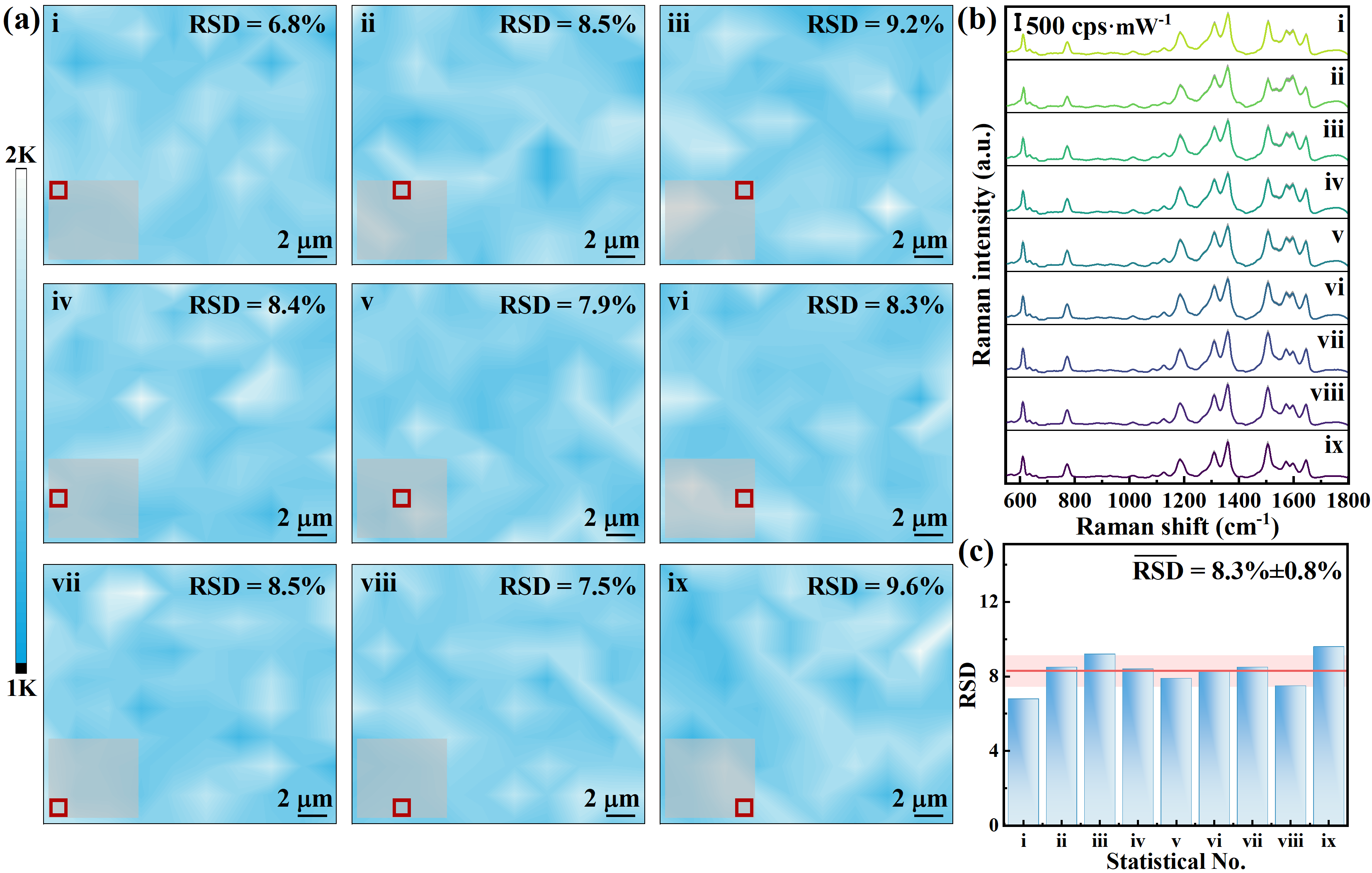


**Figure S5**. **Homogeneity of SERS performance on an Au*NP*s@1T’-MoTe_2_ nanograting.** (a) Homogeneity of 9 typical 20×20 µm^2^ regions across a 10 mm^2^ area, where the characteristic Raman shift at 1184 cm^-1^ of 10^-9^ M R6G was used for calibration. (b) Mean Raman spectra in the 9 mapping regions. (c)Mean RSD from the 9 mapping regions.


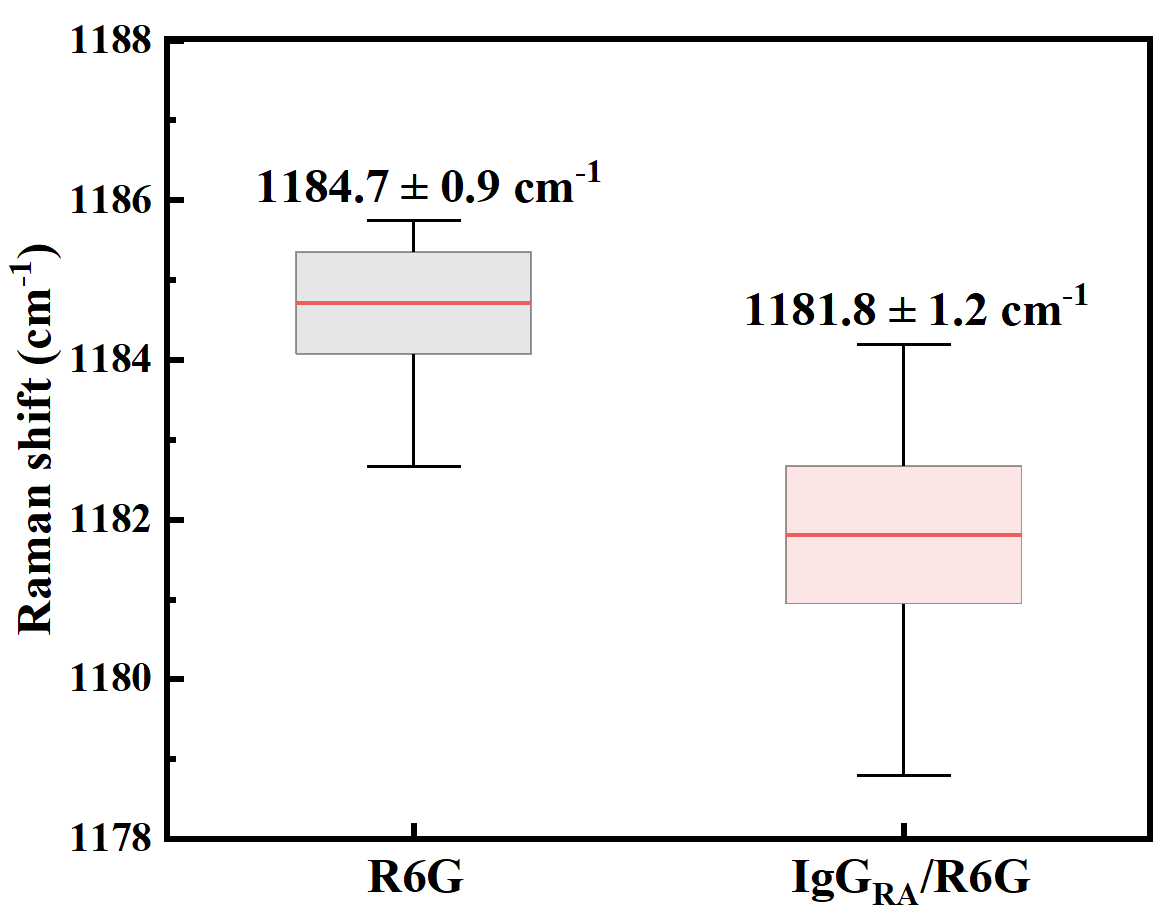


**Figure S6**. Difference in frequency shift between R6G and IgG_RA_/R6G-labeled Au*NP*s@1T’-MoTe_2_ nanogratings.


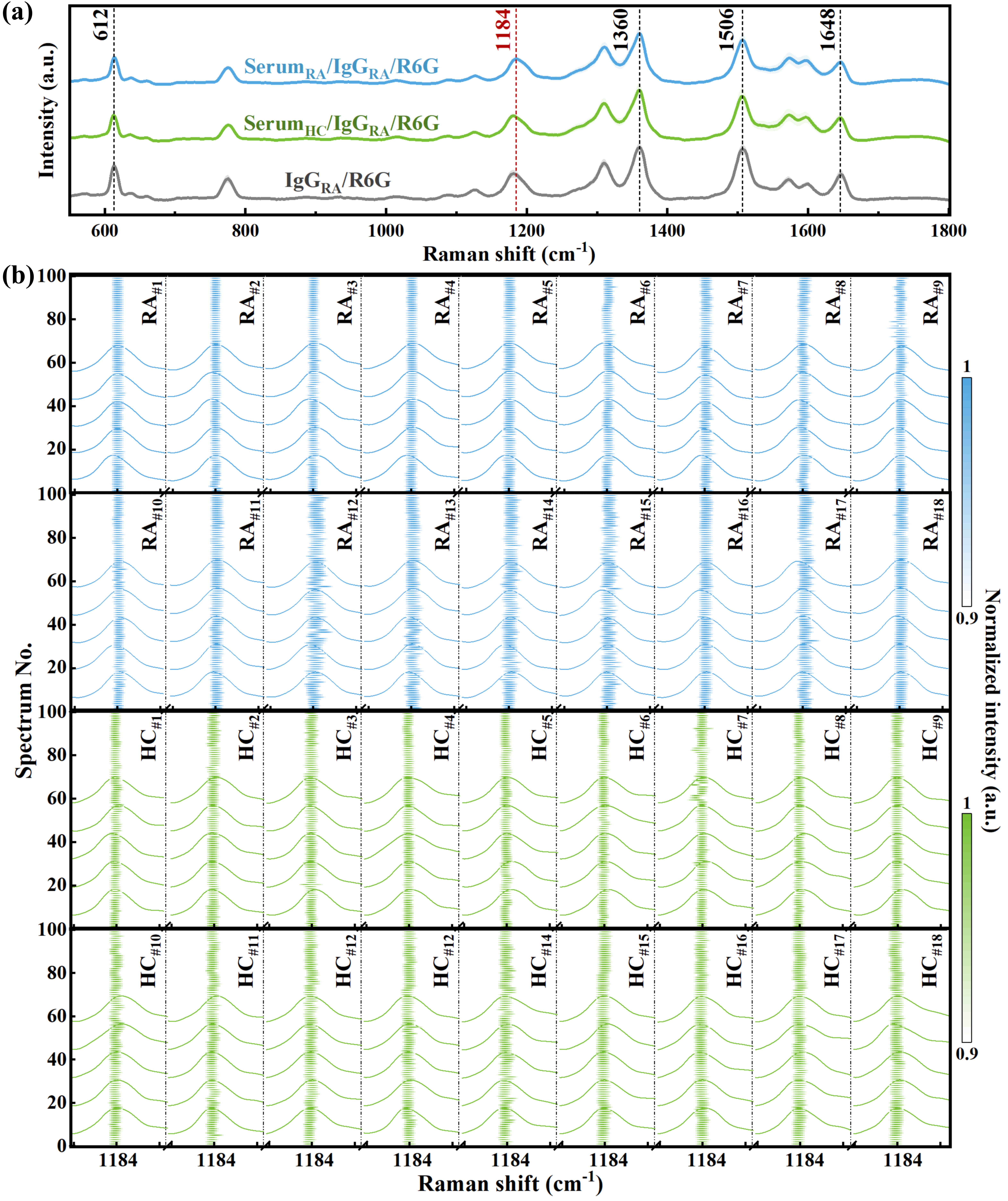


**Figure S7. Raw Raman spectra of frequency-shift dSERS immunoassay of 18 RA and 18 HC sera for dual threshold determination.** (a) Frequency shift of the characteristic Raman peak at 1184 cm^-1^ by targeting different sera. (b) Striped heatmaps with 5 typical raw Raman spectra.


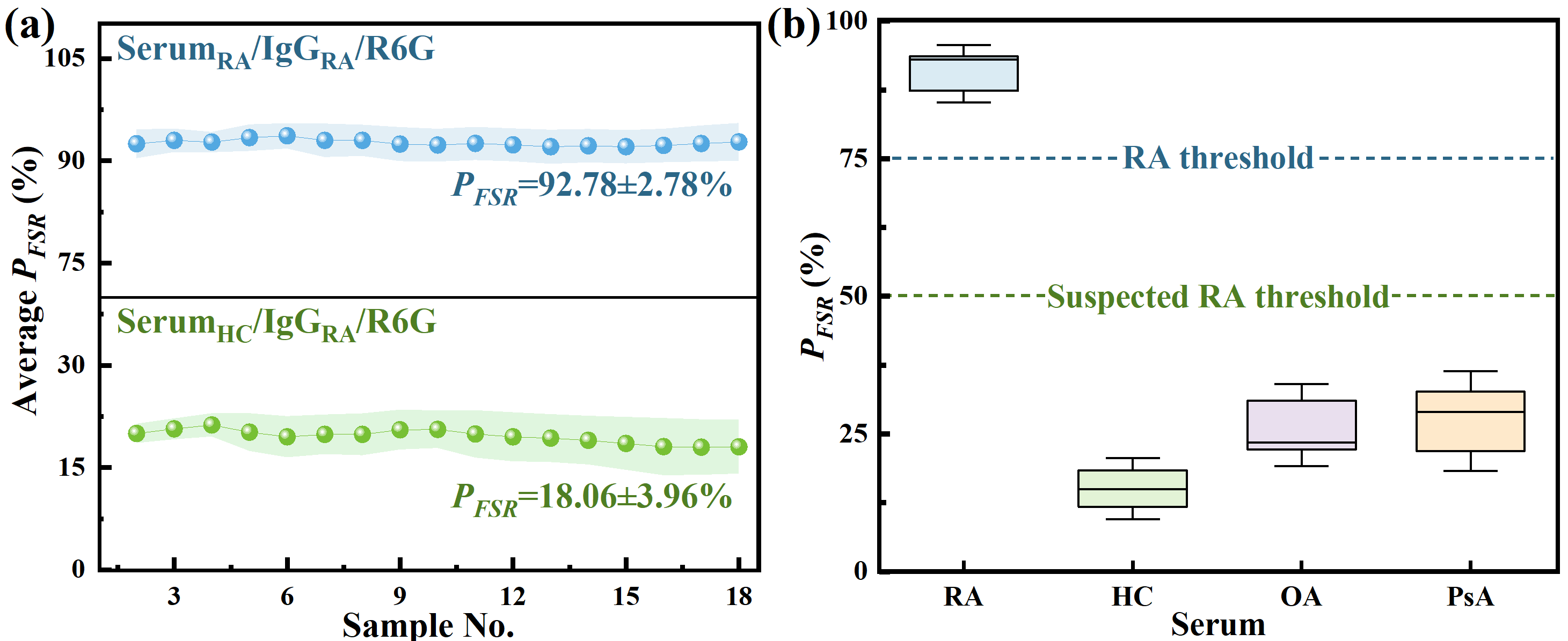


**Figure S8.** **Sample size for frequency-shift immunoassays and significance in *P_FSR_* differences between sera.** (a) Evolution of *P_FSR_* in serum_RA_/IgG_RA_/R6G and serum_HC_/IgG_RA_/R6G with sample size, where the shaded areas represent the standard deviations. (b) *P_FSR_* with significant differences between RA and non-RA sera.


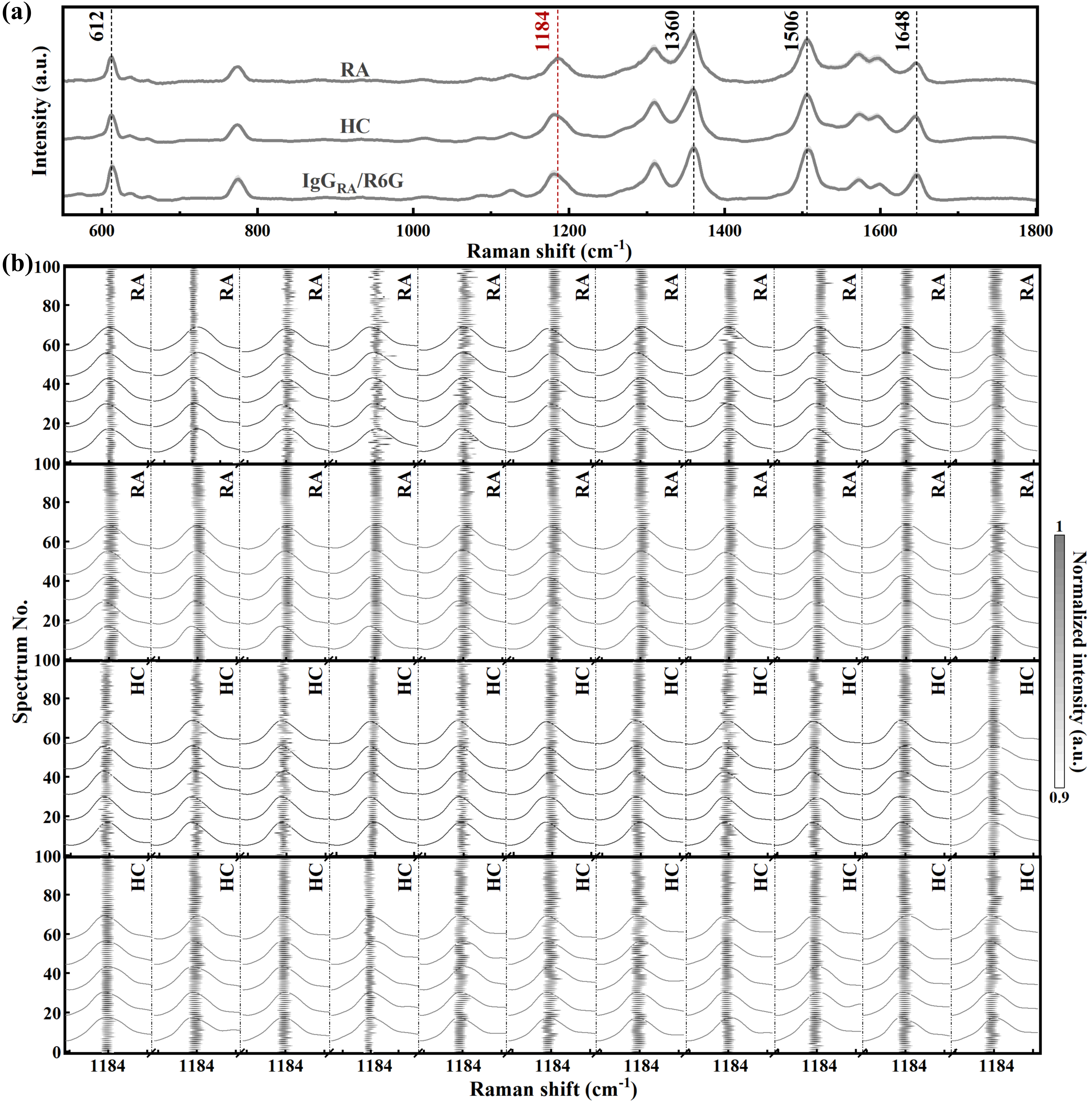


**Figure S9. Single-blind test group of frequency-shift dSERS immunoassay of 44 sera for single-blind test.** (a) Frequency shift of the characteristic Raman peak at 1184 cm^-1^ by targeting different sera. (b) Striped heatmaps with 5 typical raw Raman spectra.


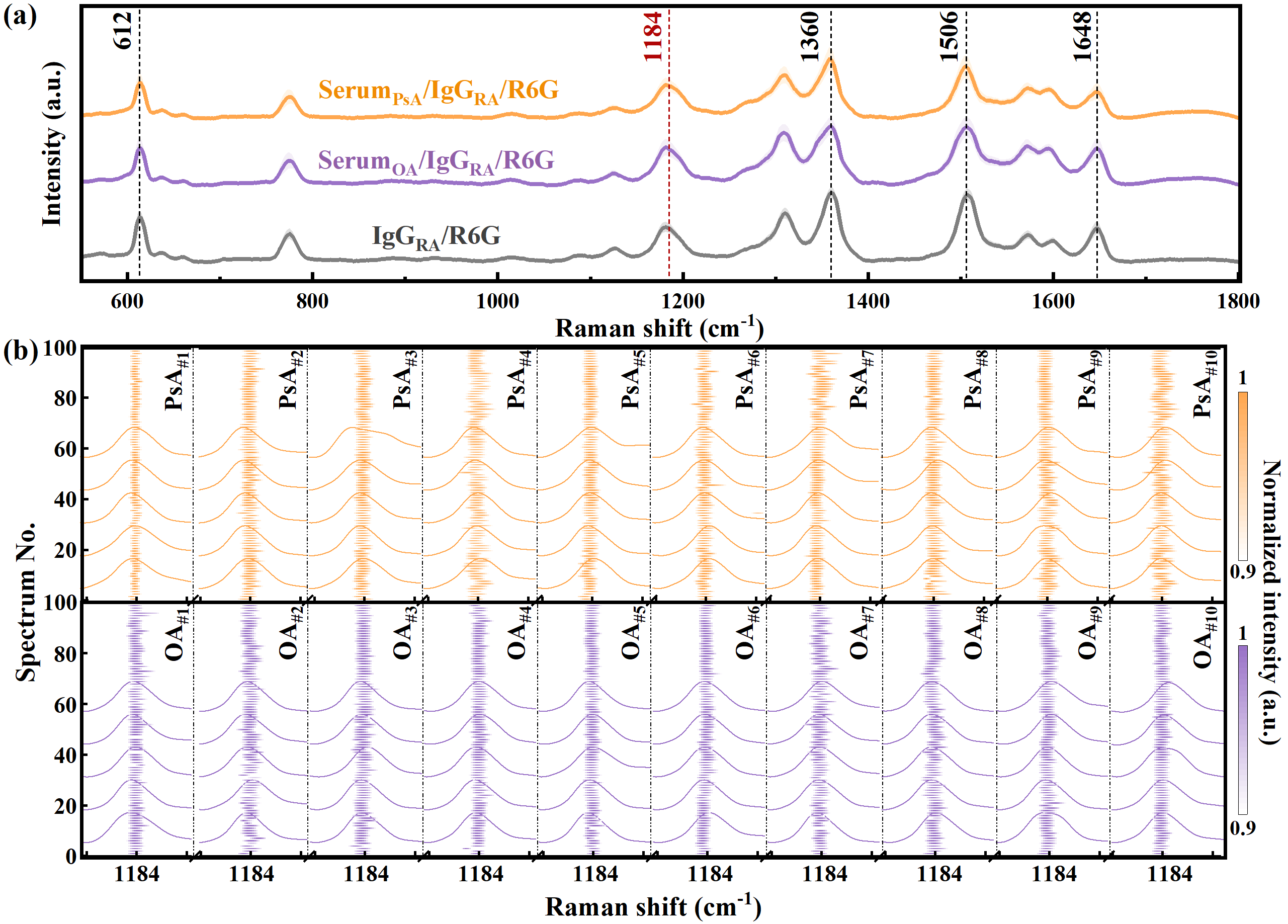


**Figure S10. Cross-reactivity validation group of frequency-shift dSERS immunoassay of 10 OA and 10 PsA sera for cross-reactivity validation.** (a) Frequency shift of the characteristic Raman peak at 1184 cm^-1^ by targeting different sera. (b) Striped heatmaps with 5 typical raw Raman spectra.


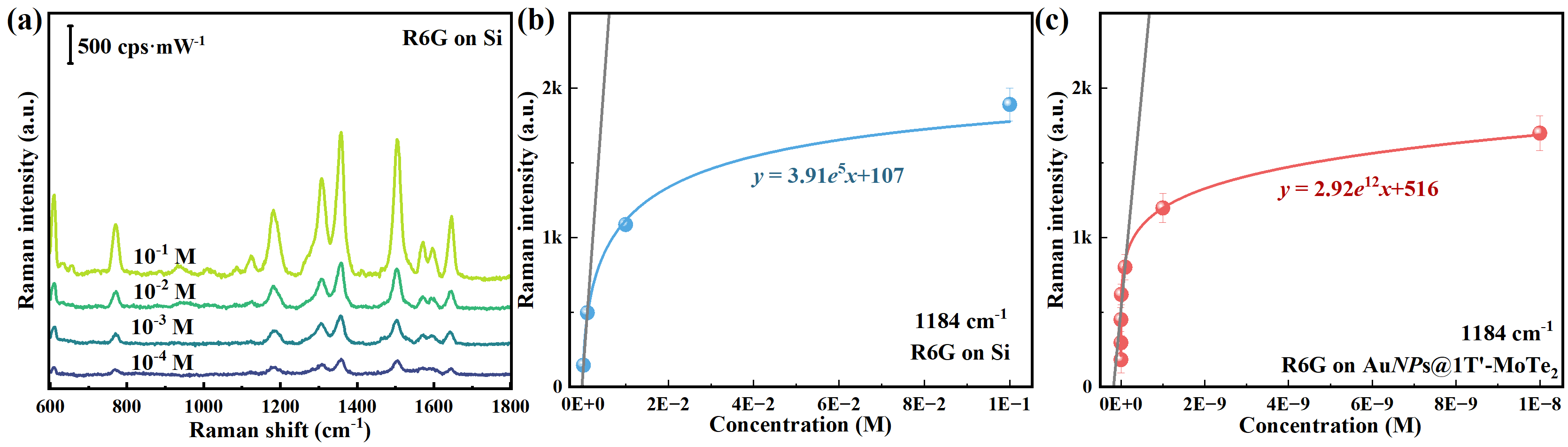


**Figure S11. Calculation of SERS performance factor.** (a) Raman spectrum of R6G molecules on Si substrates. The concentration-dependent variation in the Raman intensity of 1184 cm^-1^ peaks for R6G on (b) Si and (c) Au*NP*s@1T’-MoTe_2_ nanograting, fitted by Langmuir isotherm adsorption model.


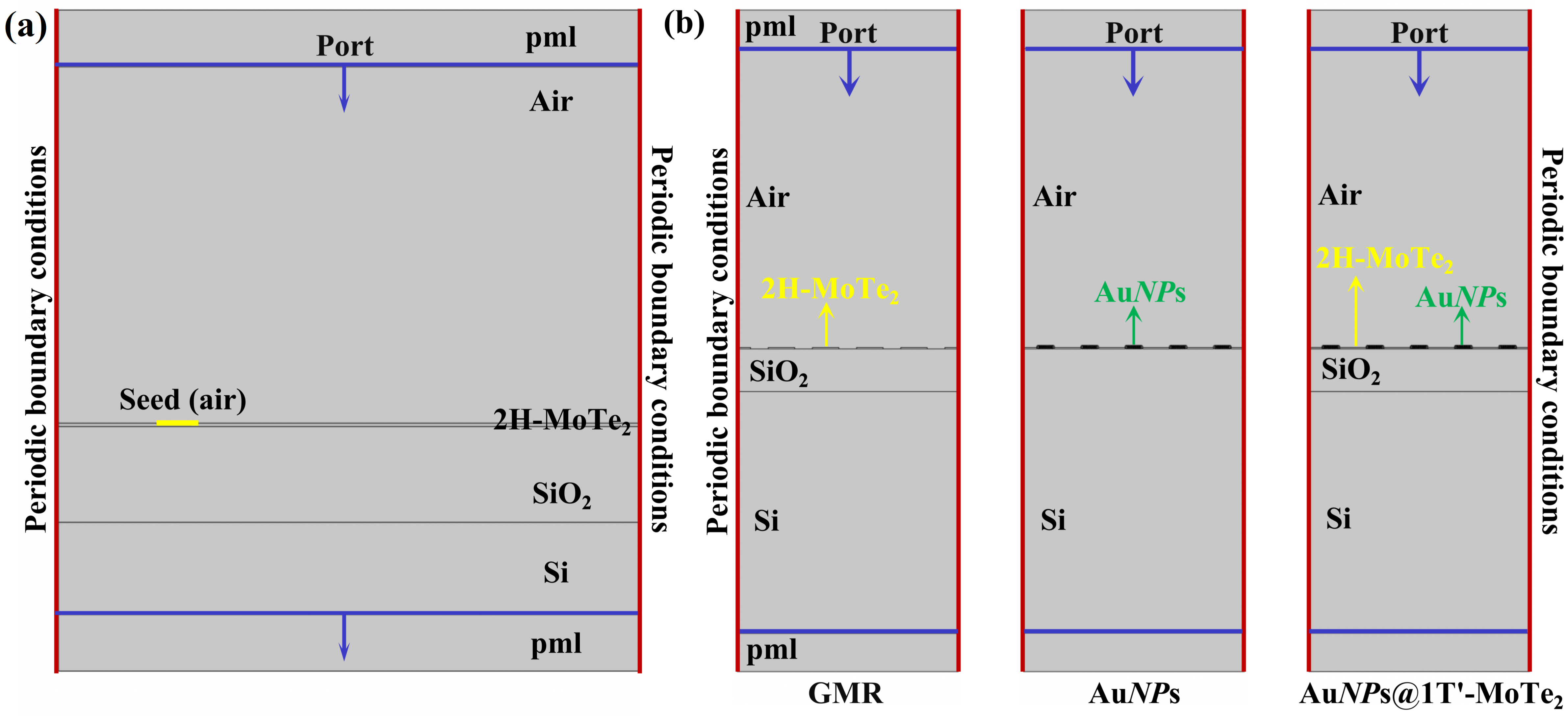


**Figure S12. Geometric models for numerical simulation.** (a) Numerical model for *fs*-LIPPT of 1T’-MoTe_2_; (b) Numerical models for on/off Fano resonances in 2H-MoTe_2_, Au*NP*s, and Au*NP*s@1T'-MoTe_2_ nanogratings.


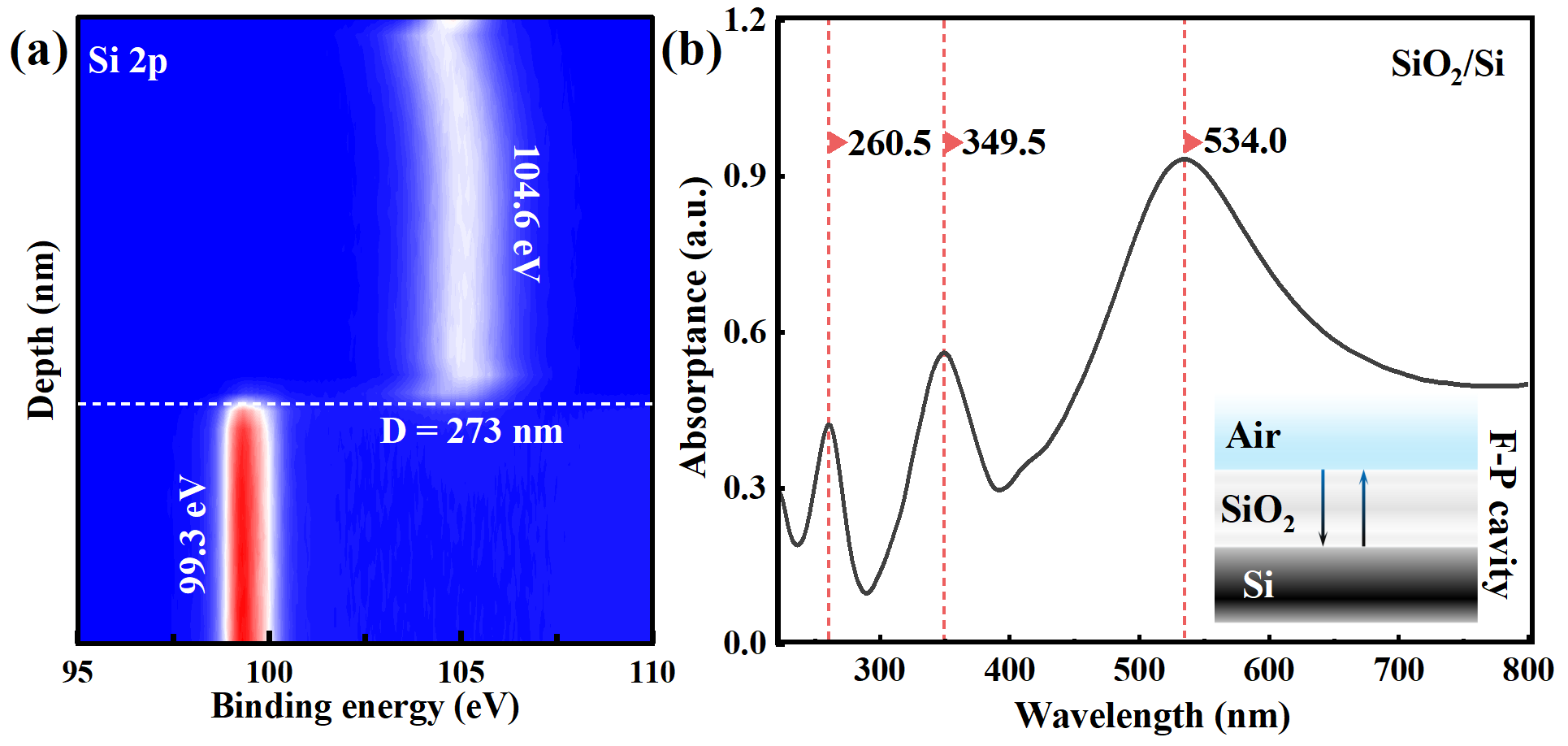


**Figure S13. Determination of thickness and refractive index of SiO_2_ interlayer.** (a) XPS spectra of Si 2p in SiO_2_/Si substrate as a function of etching depth. (b) UV-Vis absorptance spectra of SiO_2_/Si substrate.

To establish the numerical model close to the experiment, the thickness of the SiO_2_ interlayer on Si substrate was determined using depth-dependent X-ray photoelectron spectroscopy (XPS). The evolution of the Si 2p peak at 104.6 eV as a function of etching depth, obtained by continuous Ar^+^ ion etching, is shown in Figure S13a. When the etching depth reached 273 nm, the peak at 104.6 eV gradually disappeared, indicating the SiO_2_ layer etched completely. It confirmed that the thickness of SiO_2_ interlayer was 273 nm. Subsequently, the UV-Vis absorption spectrum of the SiO_2_/Si substrate was measured, as shown in Figure S13b. The absorption peaks indicated the resonance modes in the SiO_2_-interlayer Fabry-Pérot (F-P) cavity following the free-spectral range of Δ*λ*≈*λ*^2^*/2nL*, where *L* is the thickness of the SiO_2_ interlayer, *λ* is the wavelength of near the resonance peak, and *n* is the refractive index of SiO_2_. Therefore, the *n* of the SiO_2_ was calculated to be 1.94.

**Table S1**. Demographic and clinical characteristics of patients.

|  | Validation cohort | | | | Blind-test cohort | | | |
| --- | --- | --- | --- | --- | --- | --- | --- | --- |
|  | **RA (*n*=18)** | **HC (*n*=18)** | | **RA (*n*=22)** | | **OA (*n*=10)** | **PsA(*n*=10)** | **HC(*n*=22)** |
| Age, mean  (range), years | 59.32  (36-71) | 51.70  (41-67) | | 55.17  (45-71) | | 62.72  (45-71) | 56.50  (41-64) | 55.28  （32-68） |
| Female, n% | 55.5% | 50% | | 63.6% | | 70% | 50% | 45.4% |
| Duration, mean (range), years | 6.14  (1-14) | **—** | | 8.22  (3-12) | | 7.50  (3-9) | 10.7  （2-10） | **—** |
| ESR, median  (range), mm/h | 36  (4-81) | **—** | | 27  (19-119) | | 11  (3-23) | 15.81  （6-30） | **—** |
| CRP, median  (range), mg/L | 11.76  (0.48-103.9) | **—** | 5.16  (1.63-113.36) | | | 1.37  (0.5-3.36) | 8.56  (1.37-156.8) | **—** |
| RF, median  (range), IU/mL | 146.3  (1.85-1500) | **—** | | 96.3  (2.14-470) | | 3.6  (0.33-10.79) | 38.2  (0.17-360.3) | **—** |
| Anti-CCP, median  (range), U/mL | 207.32  (1.15-328.90) | **—** | | 174.89  (4.32-297.13) | | 4.78  (2.62-10.11) | 5.32  (4.06-17.29) | **—** |
| WBC, median  (range), 10^9^/L | 7.17  (2.61-11.68) | 5.97  (3.71-8.11) | | 8.5  (3.3-11.30) | | 6.56  (4.84-7.66) | 5.71  (3.20-7.24) | 5.81  (4.13-10.26) |
| DAS28, median  (range) | 4.06  (2.15-7.68) | **—** | | 3.89  (1.35-6.95) | | **—** | 2.8  (1.39-5.97) | **—** |
| Medication, No. (%) |  |  | |  | |  |  |  |
| Steroid | 12 (66.6%) | **—** | | 17 (77.3%) | | **—** | 3 (30%) | **—** |
| NSAIDs | 13 (72.2%) | **—** | | 16 (72.7%) | | **—** | 4 (30%) | **—** |
| DMARDs | 11 (61.1%) | **—** | | 14 (63.6%) | | **—** | 9 (90%) | **—** |
| Biologicals | 10 (55.6%) | **—** | | 12 (54.5%) | | **—** | 4 (40%) | **—** |
